# Modelling glucose dynamics during moderate exercise in individuals with type 1 diabetes

**DOI:** 10.1371/journal.pone.0248280

**Published:** 2021-03-26

**Authors:** Haneen Alkhateeb, Anas El Fathi, Milad Ghanbari, Ahmad Haidar

**Affiliations:** 1 Department of Biomedical Engineering, McGill University, Montreal, Canada; 2 Department of Electrical and Computer Engineering, McGill University, Montreal, Canada; 3 The Research Institute of McGill University Health Centre, Montréal, Canada; University of Milano Bicocca, ITALY

## Abstract

The artificial pancreas is a closed-loop insulin delivery system that automatically regulates glucose levels in individuals with type 1 diabetes. In-silico testing using simulation environments accelerates the development of better artificial pancreas systems. Simulation environments need an accurate model that captures glucose dynamics during exercise to simulate real-life scenarios. We proposed six variations of the Bergman Minimal Model to capture the physiological effects of moderate exercise on glucose dynamics in individuals with type 1 diabetes. We estimated the parameters of each model with clinical data using a Bayesian approach and Markov chain Monte Carlo methods. The data consisted of measurements of plasma glucose, plasma insulin, and oxygen consumption collected from a study of 17 adults with type 1 diabetes undergoing aerobic exercise sessions. We compared the models based on the physiological plausibility of their parameters estimates and the deviance information criterion. The best model features (i) an increase in glucose effectiveness proportional to exercise intensity, and (ii) an increase in insulin action proportional to exercise intensity and duration. We validated the selected model by reproducing results from two previous clinical studies. The selected model accurately simulates the physiological effects of moderate exercise on glucose dynamics in individuals with type 1 diabetes. This work offers an important tool to develop strategies for exercise management with the artificial pancreas.

## Introduction

Type 1 diabetes (T1D) is a chronic disease in which the immune system attacks and destroys the pancreatic beta cells resulting in the loss of insulin secretion [1]. Individuals with T1D need life-long insulin therapy, which requires either multiple daily injections or the use of an insulin pump that infuses insulin subcutaneously, guided by glucose measurements. A glucose sensor is a wearable device that continuously monitors blood glucose levels [2]. However, despite current technologies, individuals with T1D still spend significant amount of time outside the glucose target range, placing them at risk for devastating long-term complications such as heart attack, stroke, blindness, kidney disease, and amputation [3].

The artificial pancreas system is a promising solution for individuals with T1D. It is a closed-loop delivery system composed of a glucose sensor, an infusion pump, and a control algorithm. The artificial pancreas can be a single-hormone system that infuses insulin or a dual-hormone system that infuses insulin and glucagon [2]. The control algorithm automatically computes the hormonal doses based on glucose levels. Randomized clinical trials have shown that the artificial pancreas improves glucose control and reduces hypoglycemia [4].

Regular exercise is recommended by the American Diabetes Association [5] for its numerous beneficial effects [6], yet, around 63% of individuals with T1D are inactive [7], with the fear of hypoglycemia being the greatest barrier to exercise [8]. Even with the use of the artificial pancreas, exercise management remains a challenge [9], and new exercise control algorithms are needed to reduce the occurrence of exercise-induced hypoglycemia [10].

Enhancing the control algorithms can be accelerated by performing in-silico testing in simulation environments [2] before moving into clinical trials. Simulation environments contain virtual patients, each represented by a set of parameters used to simulate glucoregulatory models [11–14]. In order to test the safety, efficacy, and limitations of the control algorithm before moving into clinical trials, the glucoregulatory models need to incorporate the exercise effect on glucose dynamics.

A few models are reported that describe the exercise effect on glucose dynamics. In particular, Roy and Parker [15] extended the Bergman Minimal Model [16] to incorporate the effects of exercise on glucose levels. Parameter estimates in their exercise model were based on data from healthy individuals performing different exercise protocols, but the model was validated by reproducing results from a previous exercise study performed by individuals with T1D. Ewings et al. [17] modified Roy and Parker’s model, and estimated model parameters using data from individuals with T1D. Breton [18] proposed another extension of the Minimal Model using heart rate as a marker of energy expenditure. Model parameters were estimated using data from individuals with T1D undergoing exercise during hyperinsulinemic clamp protocol. Dalla Man et. al [19] incorporated an extension of Breton’s exercise model into a simulation environment of the glucose–insulin system [20]. They proposed three candidate models and performed in-silico testing to compare and validate the models. Their selected model accounts for exercise duration and intensity. A recent model by Schneider [21] has been developed by expanding the Bergman Minimal Model [16] to capture the effects of aerobic and anaerobic exercise on glucose dynamics in individuals with T1D. The model uses heart rate to distinguish between aerobic and anaerobic exercise. In-silico experiments showed that the extension model captures the effects stated within the literature. Kim et al. [22] developed a model to predict the hormonal changes during exercise. Their model was validated with experimental data where non-diabetic participants performed moderate intensity exercise for 60 min. Palumbo et al. [23] introduced some modifications to Kim’s model to simulate the effects on personal metabolic homeostasis during exercise of different durations and intensities. Hernandez et al. [24] proposed another exercise model using the percentage of maximum oxygen consumption and the percentage active muscle mass as inputs to calculate the change in hepatic glucose production, peripheral glucose uptake, and peripheral insulin uptake. Resalat et al. [25] incorporated Hernandez’s model into a model of single- and dual-hormone virtual patient population. Other exercise models have been proposed [26–29]. In all previous studies, the models were either proposed as stand-alone models and not compared to other candidate models or were not developed from real T1D clinical data.

In this paper, we proposed six physiologically-motivated variations of the Bergman Minimal Model [16] to capture the effects of moderate exercise on glucose dynamics in individuals with T1D. We estimated the parameters of the models using clinical data from individuals with T1D utilizing a Bayesian approach with Markov chain Monte Carlo methods. We compared the models based on the physiological plausibility of their parameters estimates and the deviance information criterion (DIC). We validated the performance of the selected model by replicating two published clinical studies.

## Methods

### A. Experimental data

Model parameters were estimated using data collected by Taleb et al [30]. The study included 17 adults with T1D who underwent four interventions. The study compared the efficacy of the dual-hormone (insulin and glucagon) to the single-hormone (insulin only) artificial pancreas during two types of exercise: continuous and interval. Continuous exercise was set at 60% VO_2max_ and lasted for 60 minutes. Interval exercise consisted of 2-minute alternating periods of 50% and 85% VO_2max_ for 40 minutes, with two 10-minute periods at 45% VO_2max_ at the start and end of the sessions. VO_2max_ values were obtained at the admission visit using a graded exercise test [31]. The study was approved by the IRCM Ethics Committee and conducted according to the declaration of Helsinki. All participants provided written informed consent.

**Fig 1:**
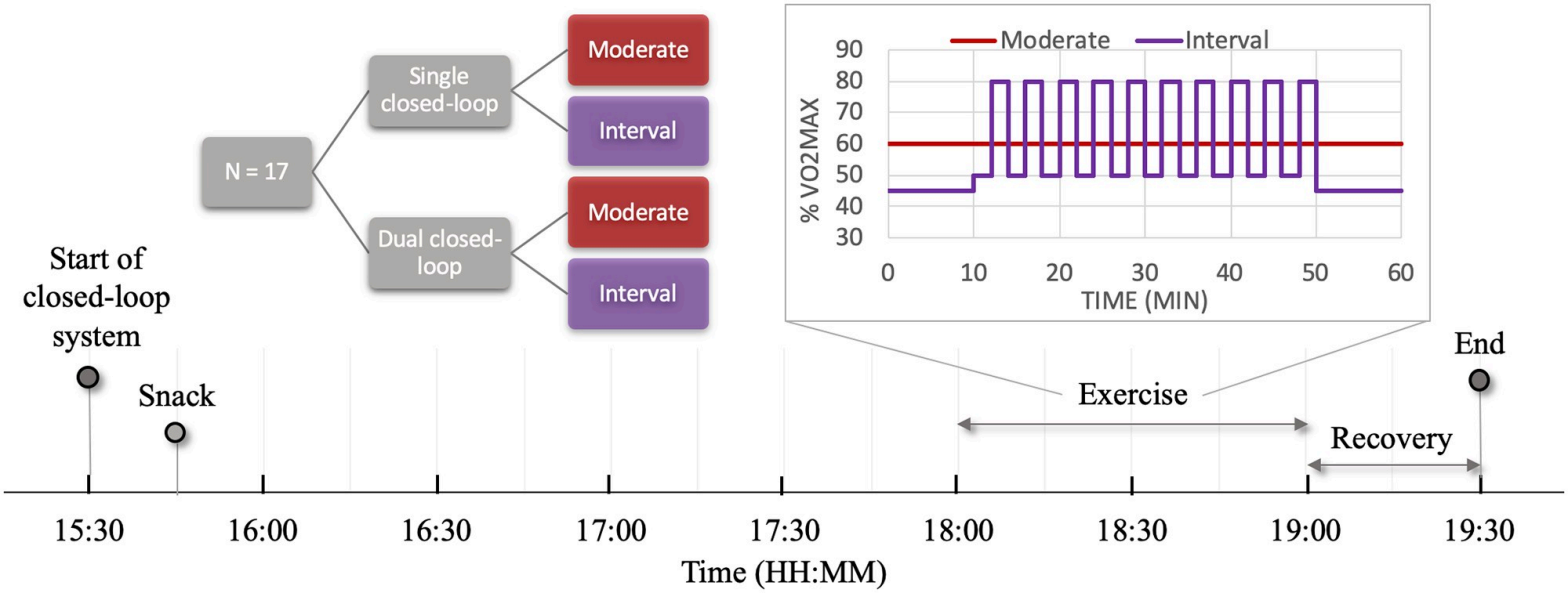
A summary of the clinical study by Taleb et al. [30].

At each intervention visit, the artificial pancreas control began at 15:30, a snack was given at 15:45, and exercise started at 18:00 followed by a 30-minute recovery period. Venous blood samples were withdrawn every 30 minutes before exercise, every 10 minutes during exercise, and every 15 minutes after exercise, totaling 14 data points for plasma glucose, plasma insulin, and plasma glucagon each per intervention. The rate of O_2_ consumption and CO_2_ production were determined from expired air samples collected using a mask during exercise. Fig 1 shows a summary of the clinical study.

**Fig 2:**
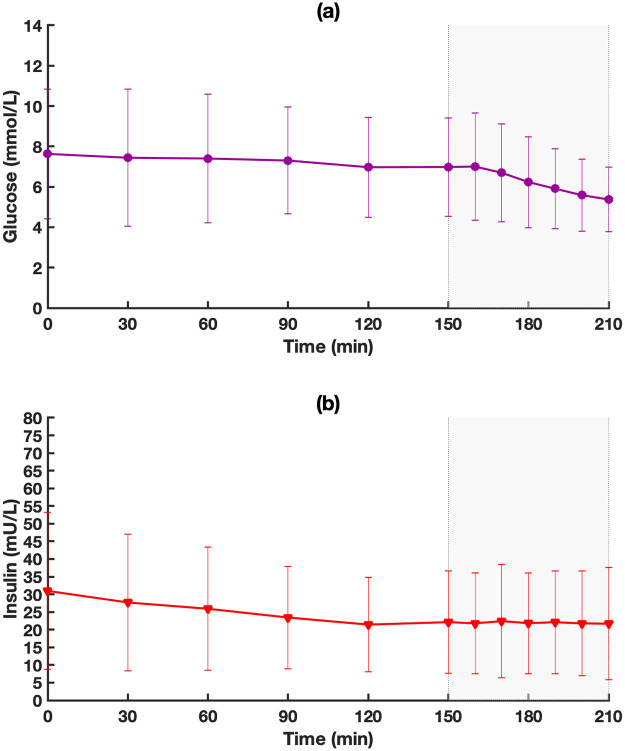
(a) Mean glucose and (b) mean insulin levels for single-hormone closed-loop during moderate exercise for the study by Taleb et al. [30]. Shaded area is exercise period. Values are mean and standard deviation (n = 11).

In this work, only data from single-hormone with moderate exercise interventions were used for our modelling purposes. Participant data that contained episodes of hypoglycemia requiring treatment during or before exercise (defined as plasma glucose <3.3 mmol/l with symptoms or <3.0 mmol/l irrespective of symptoms) were excluded and not used in the modelling. The final data set that was used for parameter estimation included data from 11 participants. Fig 2 shows mean glucose and insulin levels for single-hormone with moderate exercise data for all 11 participants used in this work. This work was approved by the McGill Ethics Committee.

### B. Model structure

The proposed models are different variations of the Bergman Minimal Model [16]. The suggested models were motivated by physiological findings and developed as sets of differential equations able to describe important physiological interactions during exercise. More specifically, the effects of glucose and insulin levels, exercise intensity, and exercise duration on glucose levels were captured. We propose six variations from the following equations structure:

$$\dot{X}(t)=-p_{2}\;X(t)+p_{3}(1+{inc}_{2}(t))Ins(t)$$ (1)

$$\dot{G}(t)=-p_{1}(1+{inc}_{1}(t))G(t)-X(t)G(t)+p_{1}\;G_{p0}$$ (2)

where

$$X(0)=\frac{p_{3}}{p_{2}}\;{Ins}_{b},$$

and

$$G(0)=G_{p0}\frac{p_{1}}{\left(p_{1}+\frac{p_{3}}{p_{2}}\;{Ins}_{b}\right)}$$

Inc_1_(t) and inc_2_(t) define the six variations, as described in Table 1, and model parameters are defined in Table 2.

**Table 1:** Proposed exercise models 1–6.

Model	*inc*_1_(*t*)	*inc*_2_(*t*)
Pre-exercise	0	0
1	e_1_	e_2_
2	0	e_1_
3	e_1_	0
4	e_1_ ∙ PVO_2max_(t)	e_2_ ∙ PVO_2max_(t)
5	e_1_ ∙ t_e_(t)	e_2_ ∙ t_e_(t)
6	e_1_ ∙ PVO_2max_(t)	e_2_ ∙ (PVO_2max_(t) + t_e_(t))

*e*_1_, *e*_2_, *PVO*_2*max*_(*t*), and *t*_*e*_(*t*) are defined in Table 2.

**Table 2:** Model parameters.

Parameter	Unit	Meaning
*G*(*t*)	mmol/L	Plasma glucose compartment
*X*(*t*)	min^-1^	Remote insulin
*Ins*(*t*)	mU/L	Plasma insulin compartment
*G*_*p*0_	mmol/L	Glucose levels at zero plasma insulin
*Ins*_*b*_	mU/L	Basal plasma insulin
*p*_1_	min^-1^	Glucose effectiveness
*p*_2_	min^-1^	Time constant for plasma insulin
*p*_3_	min^-2^/mU/L	Insulin sensitivity
**Exercise parameters**		
*inc*_1_(*t*)	unitless	Increase in glucose effectiveness
*inc*_2_(*t*)	unitless	Increase in insulin sensitivity
*e*_1_	unitless	A fixed increase in glucose effectiveness
*e*_2_	unitless	A fixed increase in insulin sensitivity
*PVO*_2*max*_(*t*)	unitless	Percentage of the maximal oxygen consumption $=\frac{{VO}_{2}(t)-{VO}_{2\;rest}}{{VO}_{2\;max}-{VO}_{2\;rest}}$, VO_2 rest_ is the VO_2_ at rest.
*t*_*e*_(*t*)	unitless	Time since the beginning of exercise $=\frac{t-t_{start}}{60\;min}$
		t_start_ is the time of the start of exercise.

#### Models 1–3

Glucose enters the muscle cells via facilitated diffusion through the GLUT4 transporters [32, 33]. Exercise increases the number of GLUT4 transporters on the cell membrane by increasing the transcription in muscle cells and increasing the translocation to the cell membrane [34, 35]. This leads to an increase in the rate of glucose uptake by two separate mechanisms. First, a rapid short-term increase in insulin-independent glucose uptake, which has been associated with muscle contractions [36, 37]. Second, a broader, longer-lasting increase in insulin-dependent glucose uptake due to the increase sensitivity to the actions of insulin in muscle cells [38, 39]. The combination of these two mechanisms on the rate of glucose uptake is additive [39–43].

Model 1 simulates the rise in the rate of glucose uptake by adding a fixed increase in insulin sensitivity and glucose effectiveness at exercise onset. Model 2 assumes that the increase in glucose effectiveness is negligible. Model 3 assumes that the increase in insulin sensitivity is negligible.

#### Model 4

As the intensity of the exercise increases, the rate of hepatic glycogenolysis decreases while the rate of glucose uptake increases [44–48]. Model 4 mimics this phenomenon by making the increase in glucose effectiveness and insulin sensitivity rise with increasing exercise intensity. The percentage of the maximal oxygen consumption (%VO_2max_) is used to quantify exercise intensity.

#### Model 5

As the duration of the exercise increases, the rate of glucose uptake increases, which results in lower glucose levels [48]. Model 5 relates the increase in glucose effectiveness and insulin sensitivity to how long the individual has been exercising.

#### Model 6

In Model 6, the increase in glucose effectiveness is dependent only on exercise, while the increase in insulin sensitivity is dependent on exercise intensity and duration.

### C. Parameter estimation and model selection

A Bayesian approach was adopted for the estimation process with the use of Markov chain Monte Carlo (MCMC) methodology [49]. A combination of prior knowledge and subject data was used to generate samples from the parameter’s posterior distributions. The median of the posterior distribution was used as a point estimate of parameter estimates.

The differential equations describing the models were solved numerically using initial conditions, model parameters, and input data from plasma insulin and plasma glucagon levels. The MCMC was implemented using WinBUGS version 1.4 [50, 51], extended by WBDiff package version 1.9.4 (MRC Biostatistics Unit, Cambridge, U.K.). The measurement errors associated with plasma glucose were assumed to be normally distributed with zero mean and coefficient of variation (CV) of 2% [52].

DIC was used to compare models based on their goodness of fit and complexity. The model with lowest DIC makes the best predictions and is considered the best performing model [53]. DIC is defined as DIC = ‘goodness of fit’ + ‘complexity’. The goodness of fit is captured via the deviance:

$$D(\bar{\theta })=-2\;\text{log}\;p(y|\theta ).$$ (3)

Complexity is measured via the estimation of the ‘effective number of parameters’:

$$p_{D}=E_{\theta |y}[D]-D{(E}_{\theta |y}[\theta ])=\bar{D(\theta )}-D(\bar{\theta })$$ (4)

where *E*_*θ*|_*y*[*D*] is the expected value of *D*(*θ*) given *y* (i.e., the posterior mean deviance) and *D*(*E*_*θ*|_*y*[*θ*]) is the deviance evaluated at the posterior mean of the parameters. The DIC is then formally defined as:

$$DIC=D(\bar{\theta })+2p_{D}=\bar{D}+p_{D}$$ (5)

The best model was chosen based on: (i) the DIC score, (ii) parameter’s identifiability and physiological plausibility, and (iii) a CV below 100%. Parameter values were used to generate virtual patients and conduct in-silico experiments.

### D. Details of model validation

For the purpose of model validation, we performed in-silico simulations to reproduce results of two previously completed clinical studies [44, 54]. We generated a virtual population of 12 individuals with T1D from the parameter sets that were estimated. Meal and insulin models used for the validation are reported in the S1 File.

#### Study 1

The protocol of the first simulated study reflected a clinical study conducted by Rabasa-Lhoret et al. [44] in 8 individuals with T1D. In this study, postprandial exercise of different intensities and durations took place 90 minutes after a breakfast of 75 g carbohydrates with various premeal insulin bolus reductions. Eight protocols were simulated as shown in Table 3.

**Table 3:** Experimental protocols for study 1 [44].

Protocol	VO_2max_	Duration	Bolus reduction
1	25%	60 min	0%
2	25%	60 min	50%
3	50%	60 min	50%
4	50%	60 min	75%
5	50%	30 min	0%
6	50%	30 min	50%
7	75%	30 min	0%
8	75%	30 min	75%

#### Study 2

The second simulated study replicated a clinical study conducted by Zaharieva et al. [54], where 17 individuals with T1D had three basal rate reductions followed by 60-min exercise sessions of 50% VO2_max_ (the exercise was divided into four 15-min bouts with 5-min rest periods in between). The insulin reductions included: 1) pump stop at exercise onset, 2) an 80% basal reduction set 90 min pre-exercise, and 3) a 50% basal reduction set 90 min pre-exercise.

#### Validation using the Hovorka model

We also performed in-silico simulation using the Hovorka model [55] to reproduce results of a published clinical study by Haidar et al. [56]. A virtual population of 30 individuals were generated. Model parameters were sampled from a prior log-normal distribution with a mean taken from Wilinska et al. [12] and parameter correlations from healthy individual data [55]. Exercise effects were implemented using the parameter sets that were estimated in this paper. The equations are listed in the appendix (section D).

## Results

### A. Parameter estimation and model comparison

For each subject, 100,000 iterations of WinBUGS were run. The last 20,000 samples were used to generate the posterior distributions. Table 4 summarizes the results for parameter estimates. All model parameters were physiologically plausible and posteriorly identifiable, with CV not exceeding 50% in all cases.

**Table 4:** Parameter estimates of Models 1–6.

Model	G_b_	p_1_ (min^-1^)	p_2_ (min^-1^)	p_3_ x10^-3^ (min^- 2^/mU/L)	e_1_	e_2_	DIC
1	54.1 (27.6–61.4)	0.0019 (0.0016–0.0024)	0.0303 (0.016–0.032)	0.014 (0.0082–0.028)	115% (103%–137%)	72% (56%–122%)	471
2	30.5 (22.1–44.6)	0.0027 (0.0022–0.0037)	0.041 (0.026–0.05)	0.015 (0.0072–0.04)	-	135% (67%–191%)	731
3	23 (18.7–46.3)	0.0025 (0.0019–0.0036)	0.033 (0.024–0.042)	0.019 (0.013–0.036)	175% (143%–244%)	-	705
4	42.3 (28.1–56.2)	0.0024 (0.0018–0.0032)	0.030 (0.022–0.037)	0.016 (0.0078–0.034)	158% (128%–200%)	108% (78%–194%)	450
5	33.1 (23.6–42.2)	0.0027 (0.0019–0.0034)	0.036 (0.018–0.050)	0.021 (0.010–0.034)	171% (156%–243%)	129% (87%–174%)	750
6	32.1 (22–51.6)	0.0021 (0.0018–0.0032)	0.031 (0.019–0.052)	0.016 (0.0091–0.027)	160% (128%–205%)	77.8% (57%–138%)	425

Values are median (interquartile range) (N = 11).

**Fig 3:**
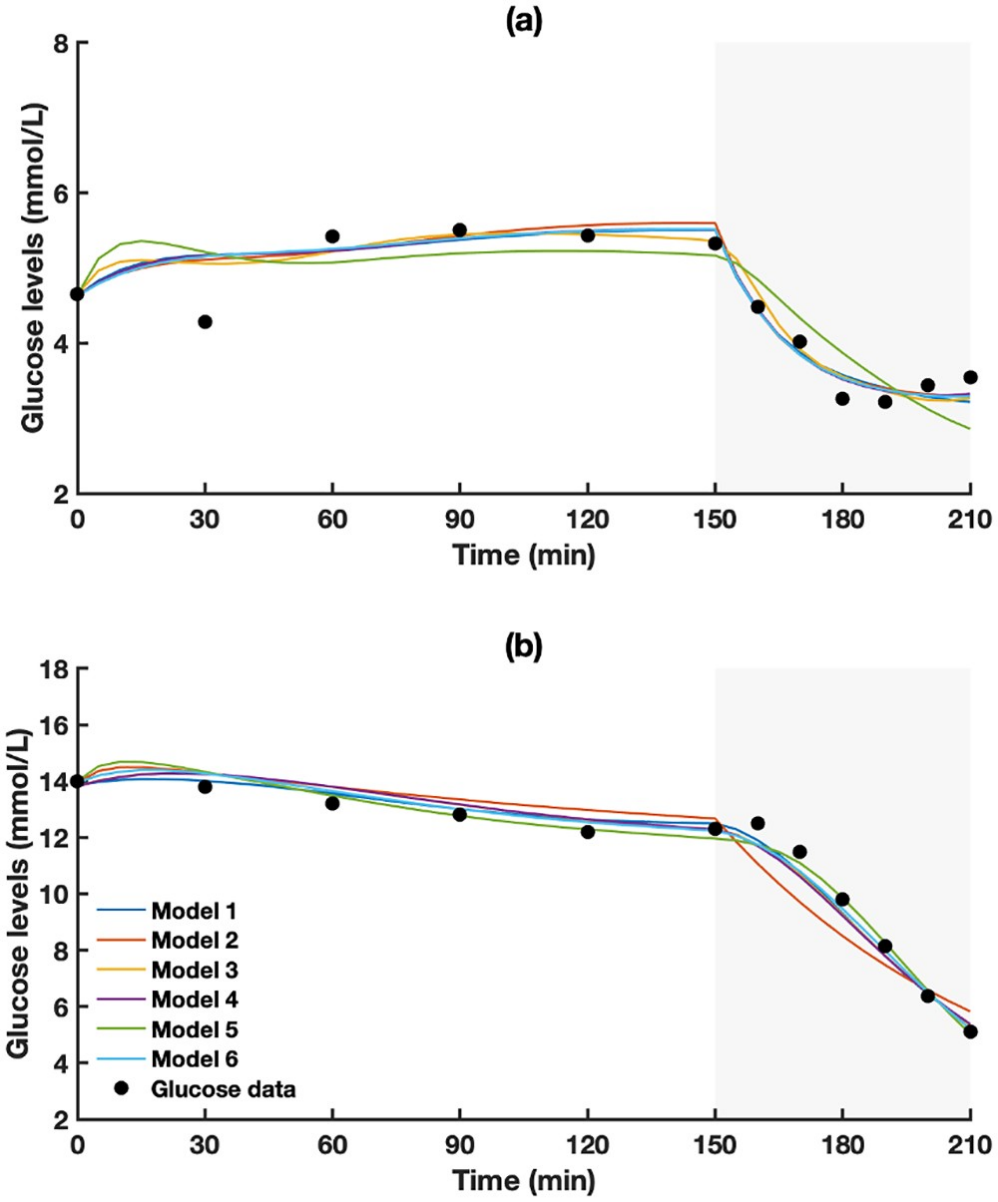
Simulated (models 1–6) vs actual glucose levels (black dots) (a) participant number 4 and (b) participant number 5. Shaded area is exercise period.

Model 1, having a fixed increase in both glucose effectiveness and insulin sensitivity, showed the DIC of 471. By neglecting the increase in glucose effectiveness, Model 2 produced a higher DIC of 731. Model 3 produced a better DIC compared with Model 2 by neglecting the increase in insulin sensitivity (731 vs 705). Model 4, incorporating the effect of exercise intensity on rate of glucose uptake, reduced DIC to 450. Model 5, incorporating the effect of exercise duration on rate of glucose uptake, did not reduce DIC compared with Model 4; its DIC was the highest with value 750 (i.e., worst performance). Combining the effect of both intensity and duration in Model 6 produced the lowest DIC of 425 (i.e., best performance). Fig 3 shows model fits on two sample occasions. Model fits for all participants can be found in the S1 Fig in S1 File.

**Fig 4:**
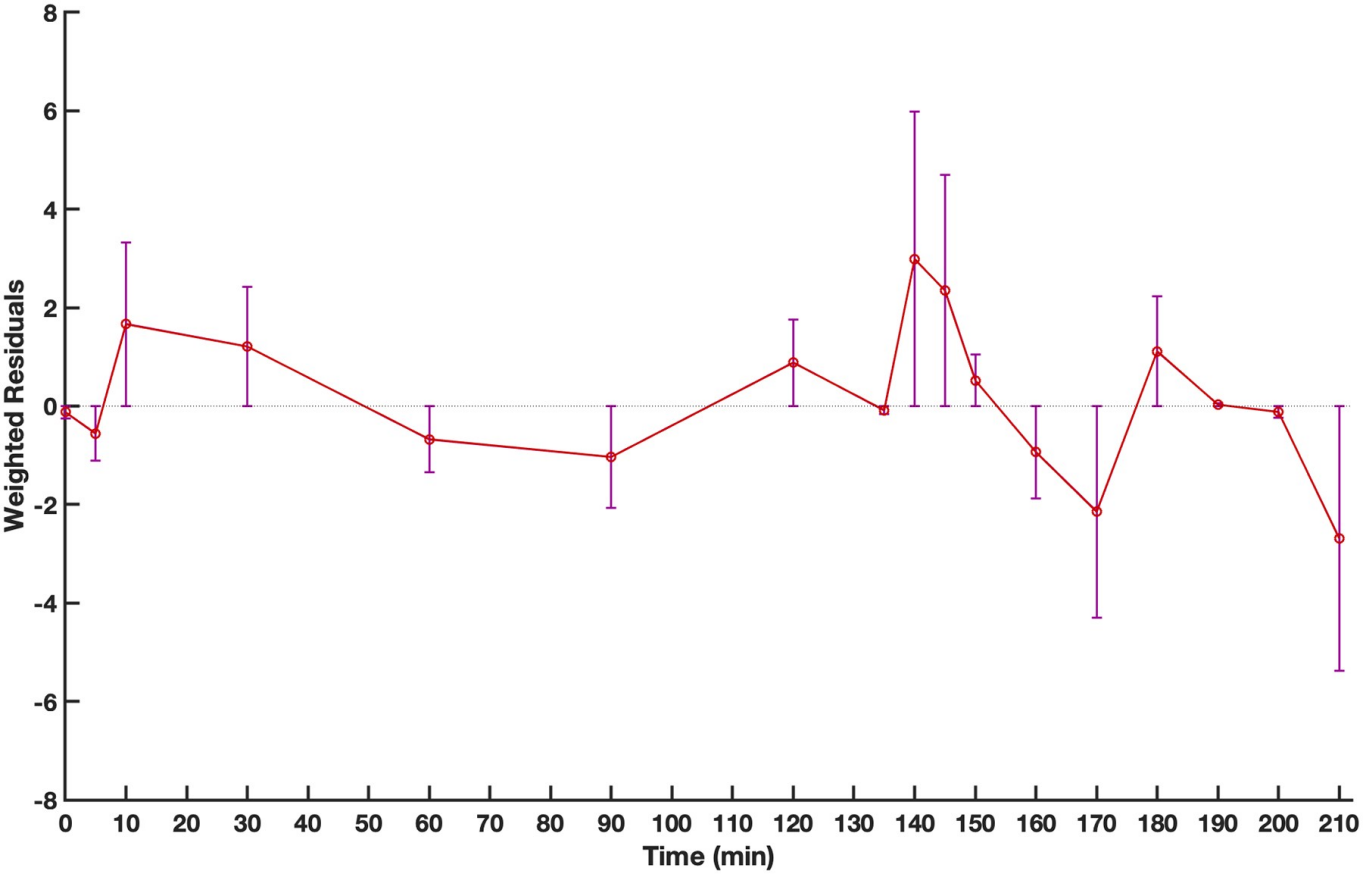
Weighted residuals for model 6’s fit. Values are median (interquartile range) (n = 11).

As a result, Model 6’s performance suggests that during exercise there is an increase in glucose effectiveness based on exercise intensity, and an increase in insulin sensitivity based on exercise intensity and duration. Additionally, all of the parameters of Model 6 were identified with good sensitivity (CV < %30). Visual inspection of Model 6’s fits confirms good performance of the model to fit the data. The weighted residuals of Model 6 are shown in Fig 4; weighted residuals indicate the ability of the model to represent the input-output relationship of the subjects without bias.

### B. Model validation

**Fig 5:**
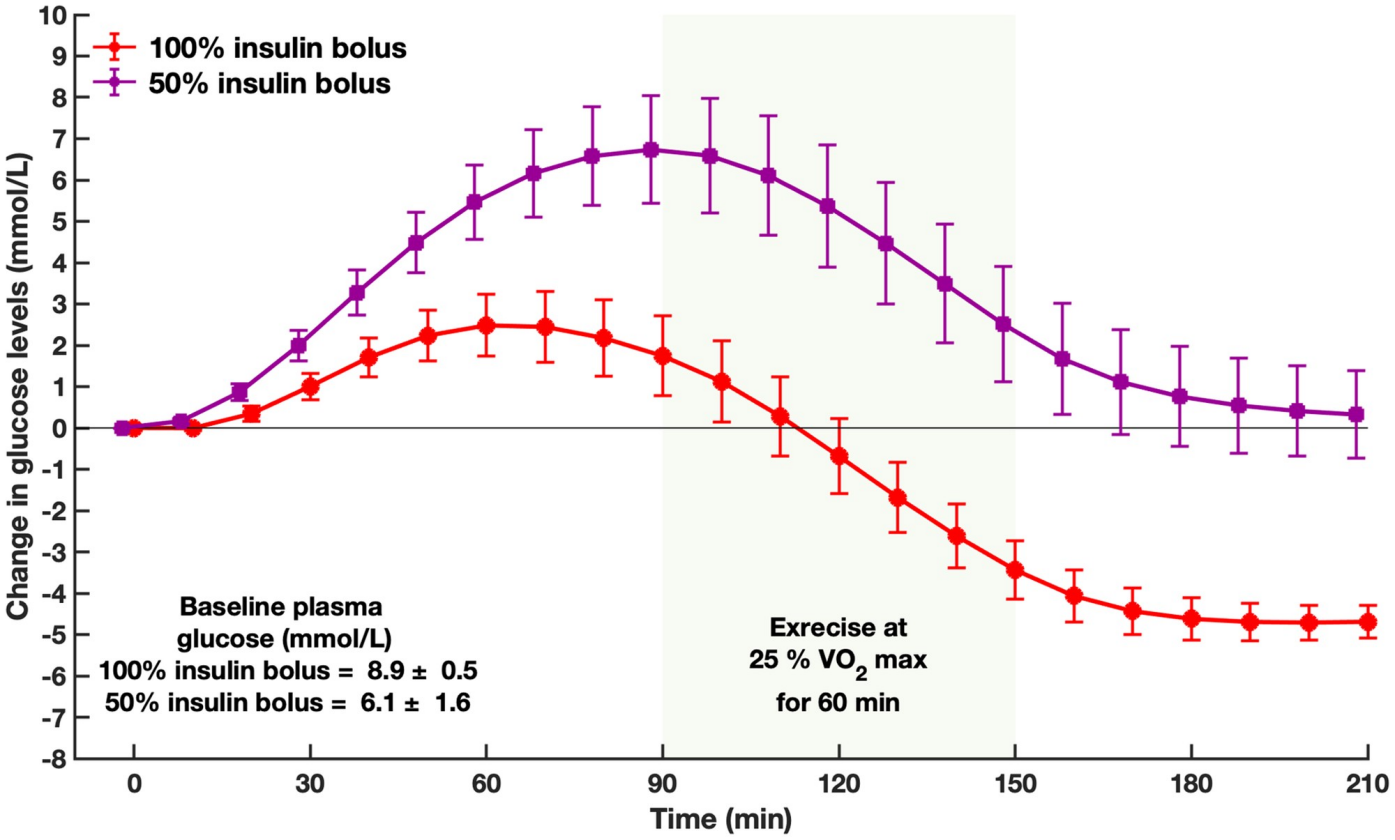
Changes in glucose levels before and during a 60-minute exercise session at 50% VO2max with 25% premeal bolus (red) 50% premeal bolus (purple). Shaded area is exercise. Values are mean and standard deviation (n = 11). Error bars represent the standard error.

The comparison between our simulation results and the results of the published clinical study of Rabasa-Lhoret et al. [44] demonstrates comparable trends in glucose levels before, during, and 60-minutes post exercise. Fig 5 shows a 50% and a 75% reduction in breakfast bolus followed by a 60-minute exercise session at 50% VO_2max_. Similar to the clinical study’s conclusion, a 75% reduction in premeal bolus resulted in a safer glycemic profile with a decreased risk of hypoglycemia compared with a 50% reduction in premeal bolus. The remaining graphs can be found in the S2–S5 Figs in S1 File.

**Fig 6:**
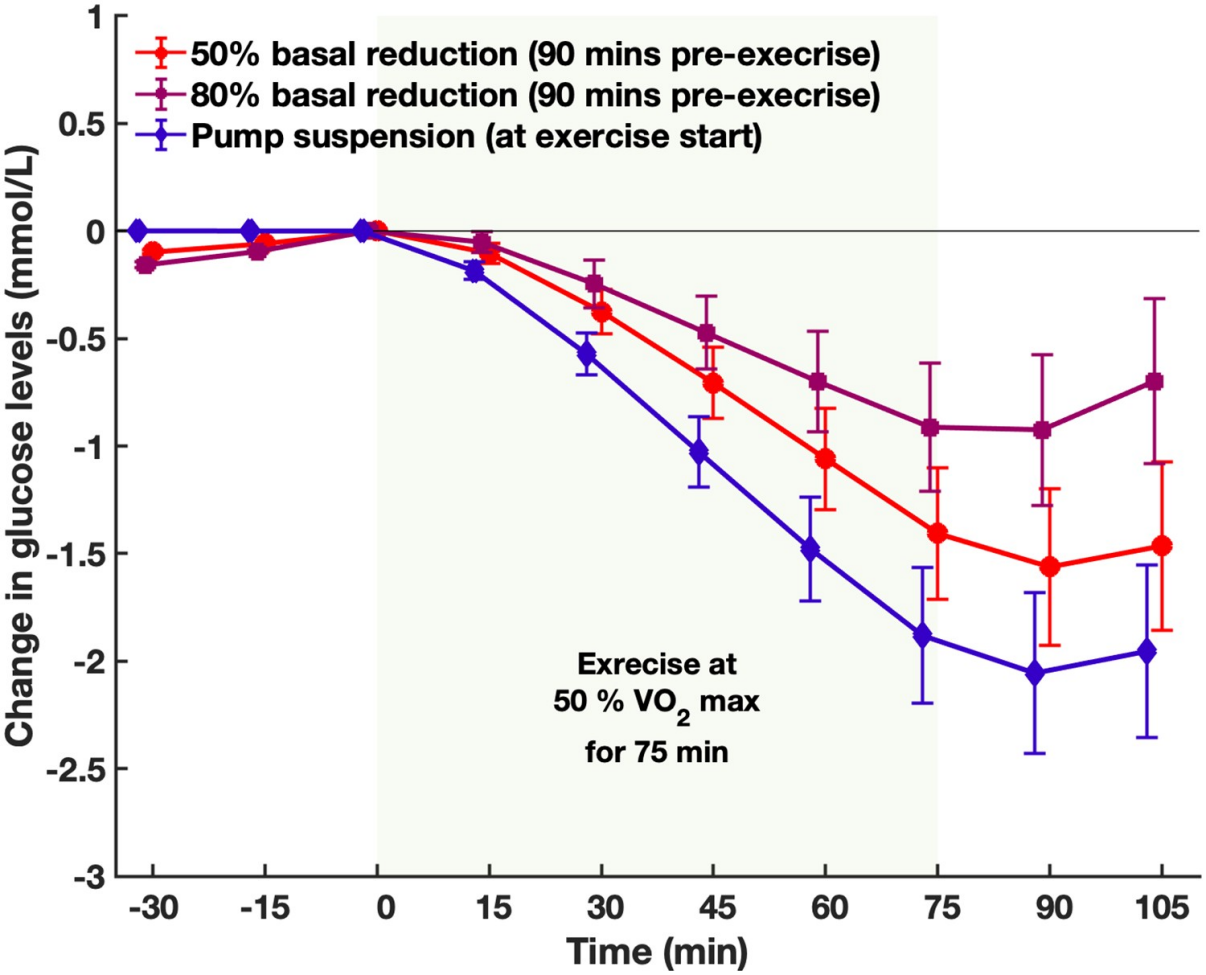
Changes in glucose levels before and during an exercise session at 50% VO2max with 50% basal reduction (red), 80% basal reduction (purple), and pump suspension (blue). Shaded area is exercise. Values are mean and standard deviation (n = 11). Error bars represent the standard error.

Similarly, simulation results demonstrate that Model 6 was able to reproduce the results obtained during the clinical study of Zaharieva et al. [54]. Fig 6 shows that by the end of exercise, a basal reduction of 80% 90 min pre-exercise showed the smallest drop in glucose levels compared with a basal reduction of 50% 90 min pre-exercise and stopping the pump at exercise onset.

The quantitative comparison between our simulation results using the Hovorka model [55] and the results of the published clinical study of Haidar et al. [56] are shown in Table 5. The outcomes are comparable, and the p values are not significant. Furthermore, it can be seen from Table 5 that the generated in-silico participants are representative of the real clinical participants.

**Table 5:** Glycemic outcomes comparison between clinical study and simulation study during the exercise period.

Outcome (%)	Clinical study	Simulation study	P value
Time spent between 4–8 mmol/L	63 (31)	66 (29)	0.41
Time spent between 4–10 mmol/L	72 (28)	74 (24)	0.36
Time spent below 4 mmol/L	0 (0 to 32)	0 (0 to 25)	0.59
Time spent below 3.3 mmol/L	0 (0 to 0)	0	0.67

Data are reported as mean (SD) or median (IQR).

## Discussion

Mathematical models that incorporate the effects of exercise on glucose dynamics play an important role in accelerating the development of safe and effective artificial pancreas systems usable in normal life conditions. In this paper, we proposed six variations of the Bergman Minimal Model [16], compared them on the basis of their physiological plausibility and DIC, and validated them against experimental data.

In people with T1D, exercise increases the rate of their glucose uptake [57]. However, specific details of how and what causes this increase is not completely understood. At least two components are responsible, an insulin-independent component and an insulin-dependent component [58]; they depend on exercise intensity, exercise duration, or a combination of both, as well as other factors. We proposed variations of the Bergman Minimal Model [16] to explore the effects of these factors. The first model simulated a fixed, exercise-induced rise in both glucose effectiveness and insulin sensitivity. Models 2 assumed a zero increase in insulin sensitivity (e_1_ = 0), and Models 3 assumed a zero increase in glucose effectiveness (e2 = 0). Model 4 related the increase in glucose effectiveness and insulin sensitivity to exercise intensity; meanwhile, Model 5 related the increase in glucose effectiveness and insulin sensitivity to exercise duration. Finally, Model 6 related the increased glucose effectiveness to exercise intensity and related the increased insulin sensitivity to exercise intensity and duration. In our system identification problem, the input to our system is exercise, which is not modified by glucose levels, and thus, this system identification problem is considered in open-loop settings. The insulin infusion rate is indeed altered by glucose levels, but this is considered an inner loop to the system. In addition, insulin infusion rate has been mostly suspended during exercise.

The outcomes of our work are consistent with findings from literature. Models 1 produced a higher DIC compared with Models 4 and 6 which suggests that adding a fixed increase on both glucose effectiveness and insulin sensitivity for the duration of the exercise is inaccurate. This agrees with two previous studies [46, 47] that demonstrated a varying rise in the rate of glucose uptake during moderate exercise. In Model 2, neglecting the increase in glucose effectiveness produced a higher DIC value. The high DIC supports the conclusion indicated by Romeres et al. [59] that the increase in insulin-independent rate of glucose uptake during moderate exercise is approximately 2–3 folds, which is an increase that cannot be neglected. By neglecting the increase in insulin sensitivity, Model 3 produced a lower DIC and improved fits compared with Model 2. This result supports the observation made by previous studies [48, 60, 61] showing that the increase in insulin-independent rate of glucose uptake is rapid at exercise onset, and quickly returns to its initial value after the end of exercise; meanwhile, the increase in insulin-dependent rate of glucose uptake is slow at exercise onset, and takes longer to returns to its initial value after the end of exercise. This is evident by the improvement of Model 3’s DIC, suggesting that during exercise, the increase in glucose effectiveness has a more-noticeable effect on glucose uptake compared with the increase in insulin sensitivity.

A particularly interesting finding in our work is related to the comparison between the models that included the effect of exercise intensity vs exercise duration. Adding the effect of exercise intensity on the increases in glucose effectiveness and insulin sensitivity produced a low DIC value and good fits as shown in Model 4, confirming findings in literature [47, 48] that higher exercise intensity results in higher glucose uptake. Duration alone, on the other hand, did not result in better fits as shown in Model 5, indicating that exercise duration has less effect on glucose uptake rates than exercise intensity. Furthermore, a combination of exercise intensity and exercise duration produced the lowest DIC and best fits as seen in Model 6, which agrees with findings from literature [48, 59]. Romeres et al. [59] showed that the rise in insulin-independent glucose uptake rate does not change for the duration of the exercise; meanwhile, the rise in insulin-dependent glucose uptake rate continues to increase as the exercise duration increases. Additionally, Nguyen et al. [48] showed that exercise intensity affects both insulin-independent and insulin-dependent rates of glucose uptake. Because of Model 6’s DIC value and good fits, it was chosen as the best model to capture the exercise-induced changes in glucose dynamics.

In-silico validation showed that Model 6 successfully predicted changes in glucose levels observed in clinical studies of moderate exercise. The simulations used virtual patients with the Model 6’s parameters estimated from the single-hormone closed-loop moderate-exercise data. The comparison between our simulated results with Model 6 and the clinical study of Rabasa-Lhoret et al. [44] demonstrated similar qualitative changes in glucose levels during and post-exercise, leading to similar clinical conclusions regarding hypoglycemia risks with different exercise intensities (25–75% VO_2max_), durations (30–60 min), and pre-meal insulin bolus reductions (25–75% reductions). However, there was some quantitative differences with both studies that are likely due to the mismatch between the meals used in the clinical study and those modelled in the simulations, as well as the mismatch in insulin absorption kinetics and insulin sensitivities between the virtual patients and those specific to the real patients recruited in the clinical study. In-silico validation using the Model 6 integrated with Hovorka model showed good quantitative results. The comparison with the clinical study of Haidar et al. [56] demonstrated no significant difference in the percentage of time spent between 4–8 mmol/L, time spent between 4–10 mmol/L, time spent below 4 mmol/L, and time spent below 3.5 mmol/L.

We used rich data to estimate model parameters including plasma glucose, plasma insulin, and oxygen uptake [30]. However, the data were still limited by certain factors. First, the duration of pre-exercise period is not long enough to accurately estimate p_1_ and p_2_. Thus, a priori values, taken from literature, were used to produce plausible values. Another limitation is lacking a direct measure of the rate of glucose uptake in our data which require complex experimental procedures with the use of tracers and varying insulin doses in order to give more information about the effects of insulin-dependent and insulin-independent rates of glucose uptake, and thus help estimate e_1_ and e_2_ more accurately. Additionally, the data did not include multiple exercise durations which could help develop models with more complex relationship instead of the linear relationship of glucose effectiveness and insulin sensitivity on exercise duration (as is the case in Model-5 and Model-6). Finally, due to lack of post-exercise data, we were not able to model early and late post-exercise period.

In this work, only the effects of moderate exercise were modeled. Interval exercise is associated with higher glucose levels than moderate exercise [9, 62]. This is evident by the data of interval exercise [30] used in this work. In most participants, glucose levels increased during exercise. The structure of our models did not allow the glucose levels to increase during the exercise period, therefore, data from interval exercise produced poor fits and high DIC values (2–3 times higher than current DICs). Thus, the validity of Model 6 was restricted to moderate exercise alone unlike other model [21]. Models that can describe other types of exercise such as interval, vigorous, and anaerobic exercise as well as post-exercise effects could be the subject of future research. Additionally, the effect of long periods of exercise (> 2–3 hours) on glucose dynamics is not completely understood. Therefore, Model 6’s performance for longer exercise durations cannot be validated yet. Furthermore, fat metabolism was not considered when developing the models because exercise took place in earlier postprandial, where carbohydrates and glycogen fulfill the demands of energy and fat metabolism is relatively very low.

The current model does not take into consideration the effects of other factors, such as age and fitness levels as seen in other models [23]. Literature show differences between adults and adolescents in regard to the immediate effect of exercise [63] as a result of the higher growth hormone levels in adolescents [64], which are known to be an opposing effect to insulin action [65, 66], as well as the difference in muscle mass and insulin sensitivity between the two groups [67]. Furthermore, findings show that individuals with good fitness level seem to be more prone to exercise-induced hypoglycemia [68] because of their higher insulin sensitivity. Finally, our model’s performance was not compared to other models from literature due to data and other limitations. This could be the subject of future research.

The new exercise model proposed in this work was developed from real T1D clinical data and was compared with other models that simulate different physiological phenomena experienced by individuals with T1D during moderate exercise.

## Conclusion

Developing a model that is able to capture the changes in glucose dynamics during exercise is one of the challenges in improving closed-loop systems [69]. Such models continue to be developed to help build a safe and effective artificial pancreas usable in normal life conditions. The new exercise model proposed in this work can be incorporated in a simulation environment, enabling us to perform in-silico testing that may result in better glycemic control during moderate exercise, and reducing the events of exercise-induced hypoglycemia.

## Supporting information

S1 File (DOCX)
