# Supplementary material for: Modelling glucose dynamics during moderate exercise in individuals with type 1 diabetes
Source: PLoS One. 2021 Mar 26;16(3):e0248280. doi: 10.1371/journal.pone.0248280 (PMC7996980; doi:10.1371/journal.pone.0248280)
Supplement: S1 File — (DOCX) [file pone.0248280.s001.docx]

1. **Basic Description of Monte Carlo Markov Chain Methods**

Monto Carlo Markov Chain (MCMC) methods are a class of algorithms that allow sampling from posterior Bayesian distributions. It creates a Markov chain that converges to the posterior distribution under fairly general conditions [1]. The Metropolis-Hasting algorithm is an approach to generate the Markov chain [2]{Gilks, 1998 #369}. Basic steps of the Metropolis-Hasting algorithm applied to present computational problem are given below.

Let the vector $Y=(Y\left( 0 \right),\ldots,Y(m-1)$) represents the measurements of glucose levels, and $U$ represents the vector of individual parameters. The MCMC is used to obtain the joint posterior distribution of all unobserved stochastic variable $U$ conditional on the observed data $Y$, i.e. $P(U|Y)$. The steps are as follows:

1. Define a Markov chain $\theta^{(i)}, i=1,2,\ldots$, as $\theta^{(i)}=(U^{\left( i \right)})$
2. Initialize the Markov chain $\theta^{(1)}$ by setting suitable initial values for each of the stochastic variables, e.g., $p_{1}, p_{2}, \ldots.$etc.
3. Propose a new state of the Markov chain $\bar{\theta}^{(i)}$ that depends only on the previous state $\theta^{(i)}$.
4. Solve numerically the differential equations of the Radziuk/Mari model with accompanying initial conditions and individual glucose fluxes as defined in the proposed state $\bar{\theta}^{(i+1)}$.
5. The proposed state $\bar{\theta}^{(i+1)}$ is accepted, i.e.${\theta^{(i+1)}= \bar{\theta}}^{(i+1)}$, if $\alpha$, drawn from the standard uniform distribution, satisfies

$$\alpha< \frac{P\left( \bar{\theta}^{\left( i+1 \right)} | Y \right) f\left( \theta^{\left( i+1 \right)} \right|\bar{\theta}^{\left( i+1 \right)})}{P\left( \theta^{\left( i+1 \right)} | Y \right) f\left( \bar{\theta}^{\left( i+1 \right)} \right|\theta^{\left( i+1 \right)})}= \frac{P\left( Y | \bar{\theta}^{\left( i+1 \right)} \right) P(\bar{\theta}^{\left( i+1 \right)}) f\left( \theta^{\left( i+1 \right)} \right|\bar{\theta}^{\left( i+1 \right)})}{P\left( Y | \theta^{\left( i+1 \right)} \right) P(\theta^{\left( i+1 \right)}) f\left( \bar{\theta}^{\left( i+1 \right)} \right|\theta^{\left( i+1 \right)})}$$

where $f$ is known as a proposal or jumping density [2-4]. $P\left( Y | \theta^{\left( i+1 \right)} \right)$ and $P\left( Y | \bar{\theta}^{\left( i+1 \right)} \right)$ are calculated from the Bayesian likelihood function and $P(\theta^{\left( i+1 \right)})$ and $P(\bar{\theta}^{\left( i+1 \right)})$ are calculated from the Bayesian prior distribution. If the proposed new state is rejected, then the next state is the same as the current state, i.e.$\theta^{(i+1)}=\theta^{(i)}$

1. Go to Step 3.

The Markov chain converges to the joint posterior distribution after a sufficient number of samples, and all subsequent samples are considered as posteriors of the distribution. Point estimates of unknown parameters are then inferred from the medians of the posterior distributions.

1. **Parameter’s A-Priori**

**Table S.1: Parameters’ a-priori**

| **Parameter** | **G_b_ (mmol/L)** | $\mathbf{p}_{\mathbf{1}}$  **(min^-1^)** | $\mathbf{p}_{\mathbf{2}}$  **(min^-1^)** | $\mathbf{p}_{\mathbf{3}}$ **x10^-3^**  **(min^- 2^/mU/L)** | $\mathbf{e}_{\mathbf{1}}$ | $\mathbf{e}_{\mathbf{2}}$ |
| --- | --- | --- | --- | --- | --- | --- |
| Value | 20 (0.2) | 0.00247 (0.1) | 0.0302 (0.1) | 0.045 (0.1) | 2 (0.1) | 2 (0.1) |
| Values are mean (variance) (N=11) | | | | | | |

1. **The Pharmacokinetics of Subcutaneous Insulin Absorption Model**

In the simulation, we employed a two-compartment model to describe the pharmacokinetics of subcutaneous insulin absorption [5], and the meal-related glucose appearance [6]. The rate of appearance of glucose $R_{a}$ (mmol/L/min) and the plasma insulin concentration $I_{p}$ (mU/L) are:

$R_{a}\left( t \right)=K_{m}\frac{U_{m}}{\tau_{m}^{2}} t e^{-\frac{t}{\tau_{m}}}$ (1)

$I_{p}\left( t \right)=\frac{{10}^{3}}{K_{\mathrm{MCR}}\omega}\frac{U_{\mathrm{bo}}}{\tau_{i}^{2}} t e^{-\frac{t}{\tau_{i}}}$ (2)

where $U_{m}$ (g) is the amount of consumed carbohydrates in the meal ($U_{\mathrm{bo}}$), U is the amount of insulin bolus, $\omega$ (kg) is the patient weight, and the remaining parameters are defined in Table S.2.

**Table S.2: Parameters definitions**

| **Parameter** | **unit** | **Description** | **Value** |
| --- | --- | --- | --- |
| $K_{m}$ | mmol/L/g | Constant of proportionality (including bioavailability of carbohydrates and the glucose volume of distribution) | $\frac{25}{\omega}$ |
| $\tau_{m}$ | min | Time to peak of the carbohydrate absorption | 40 |
| $K_{\mathrm{MCR}}$ | L/kg/min | Metabolic clearance rate | 0.02 |
| $\tau_{i}$ | min | Time to peak of insulin absorption | 70 |

1. **Integration of the Exercise Model with the Hovorka Model**

In the simulation, we integrated our exercise model with the Hovorka model [7] as shown here:

| $\dot{x}_{1}\left( t \right) = -k_{a1} x_{1}\left( t \right)+k_{a1}S_{t} \left( 1+\mathrm{inc}_{2}\left( t \right) \right) Ins(t)$ | (3) |
| --- | --- |
| $\dot{x}_{2}\left( t \right) = -k_{a2} x_{2}\left( t \right)+k_{a2}S_{d} \left( 1+\mathrm{inc}_{2}\left( t \right) \right) Ins(t)$ | (4) |
| $\dot{x}_{3}\left( t \right) = -k_{a3} x_{3}\left( t \right)+k_{a3}S_{e} Ins(t)$ | (5) |
| $\dot{Q}_{1}\left( t \right) = -\left[ \frac{F_{01}}{V G\left( t \right)}+x_{1}\left( t \right) \right]Q_{1}\left( t \right)+k_{12}Q_{2}\left( t \right)+EGP$ | (6) |
| $\dot{Q}_{2}\left( t \right) = x_{1}\left( t \right)Q_{1}\left( t \right)-\left[ k_{12}+x_{2}\left( t \right) \right]\left( 1+\mathrm{inc}_{1}\left( t \right) \right)Q_{2}\left( t \right)$ | (7) |
| $G\left( t \right)=Q_{1}\left( t \right)/V$ | (8) |
| $EGP=\left\{ \begin{aligned} \mathrm{EGP}_{0}\left( 1-x_{3}\left( t \right) \right) : x_{3}\left( t \right)<1 \\ 0 : x_{3}\left( t \right)\geq1 \end{aligned} \right.$ | (9) |

where $x_{1}$, $x_{2}$, and $x_{3}$ (1/min) are the remote effects of insulin on glucose distribution, glucose disposal, and the endogenous glucose production, respectively, $k_{a1}$, $k_{a2}$, and $k_{a3}$ (1/min) are time constants, $S_{t}$ and $S_{d}$ (1/ (min mU/l)) are the insulin sensitivities of glucose distribution and glucose disposal, respectively, and $S_{e}$ (1/(mU/l)) is the insulin sensitivity of endogenous glucose production. $Q_{1}$, $Q_{2}$ (µmol/kg) represent the masses of glucose in the accessible (where glycemia measurements are made) and non-accessible compartments, respectively. $k_{12}$ (1/min) is the transfer rate parameter, $F_{01}$ (µmol/kg/min) is the non-insulin dependent glucose utilization, and ${EGP}_{0}$ (µmol/kg/min) is the endogenous glucose production extrapolated to zero insulin concentration. G (mmol/L) is the plasma glucose concentration and V (ml/kg) is the glucose distribution volume.

1. **Supplementary Figures**

| Participant 1**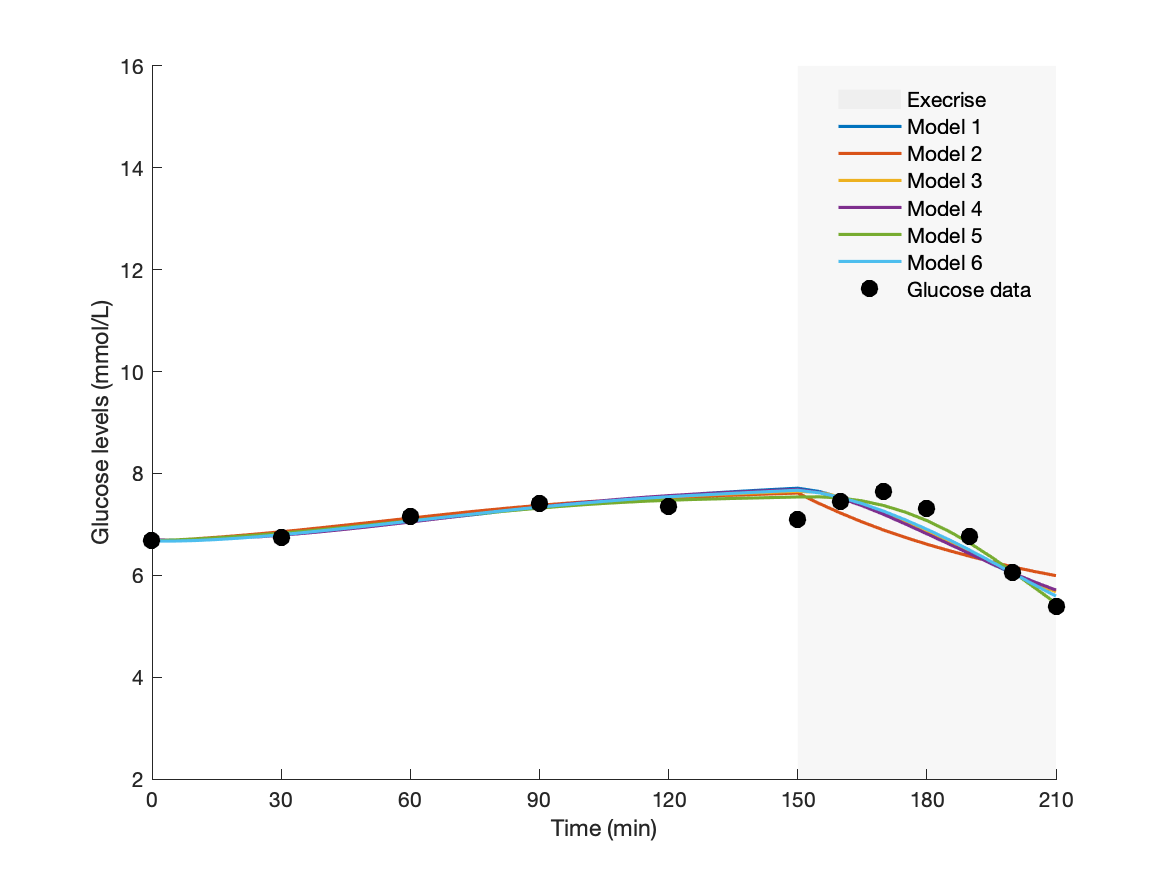** | Participant 2**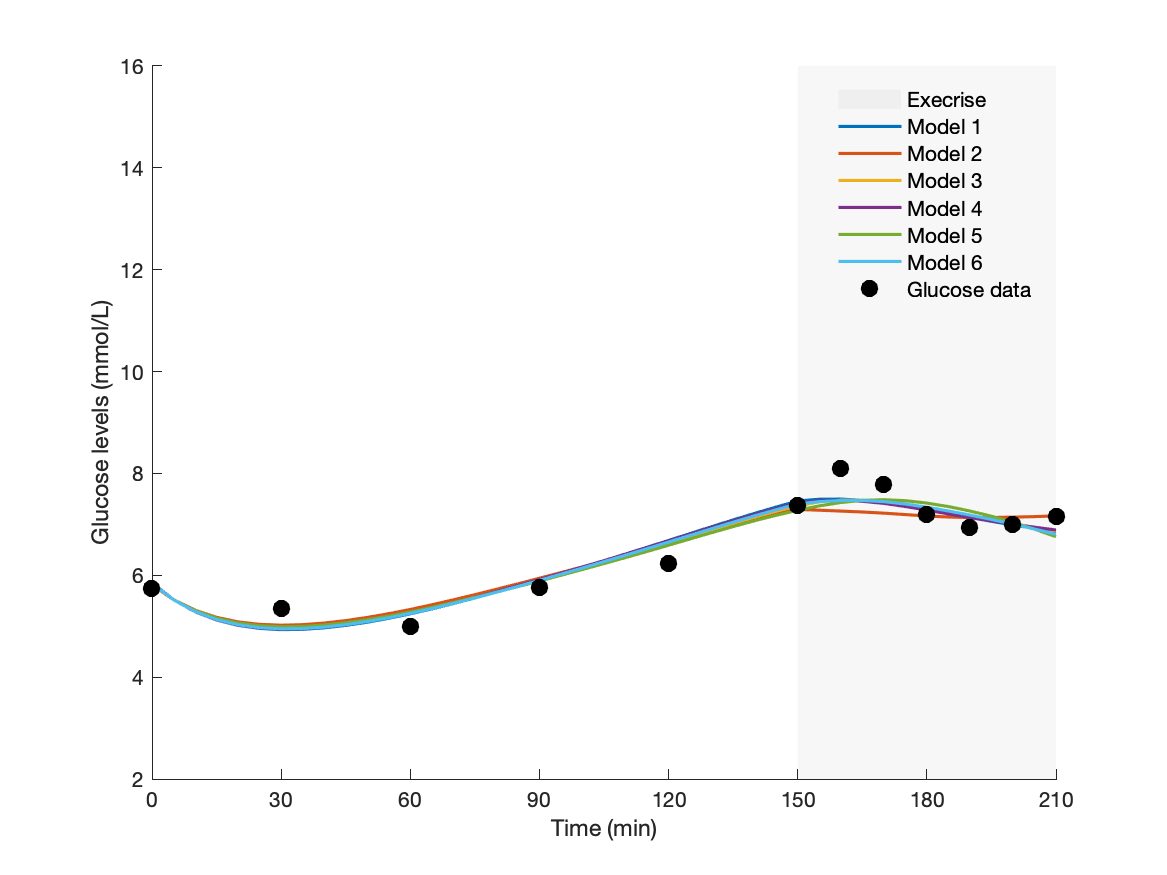** |
| --- | --- |
| Participant 3**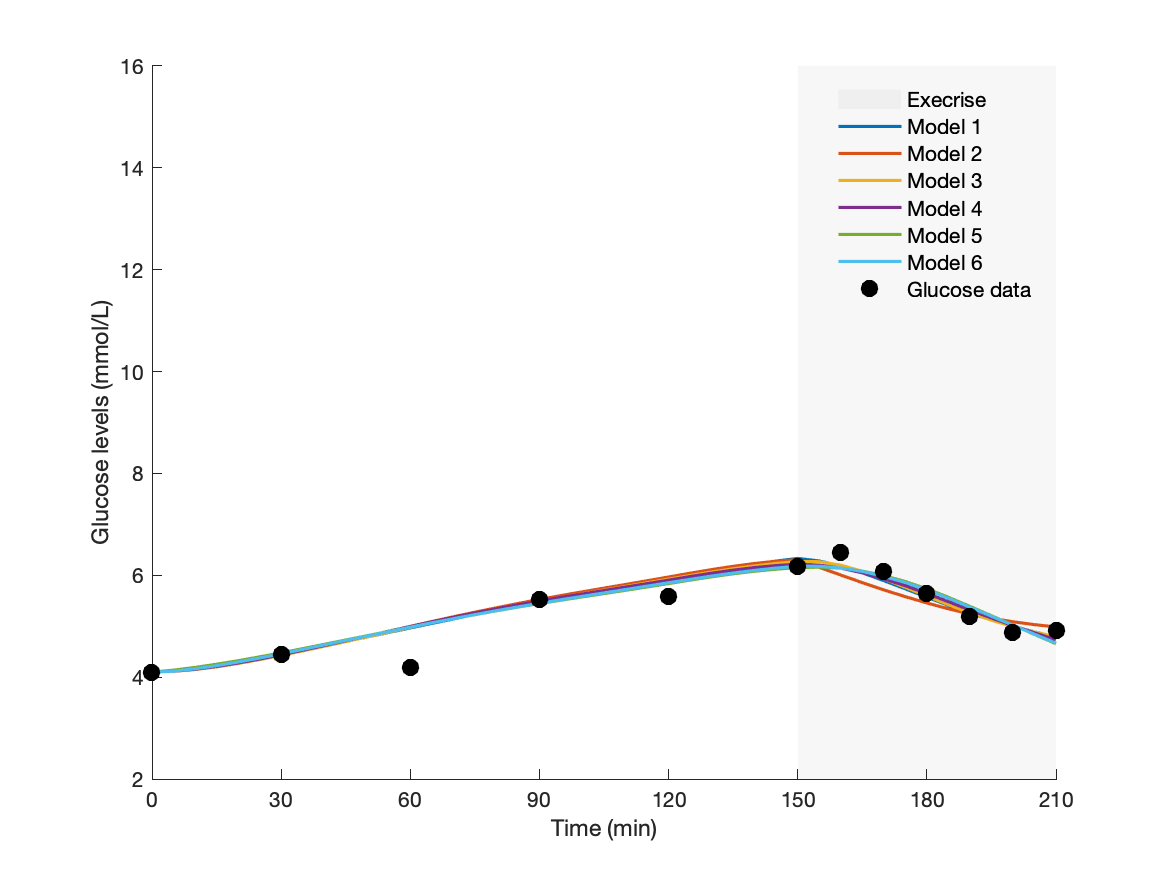** | Participant 4**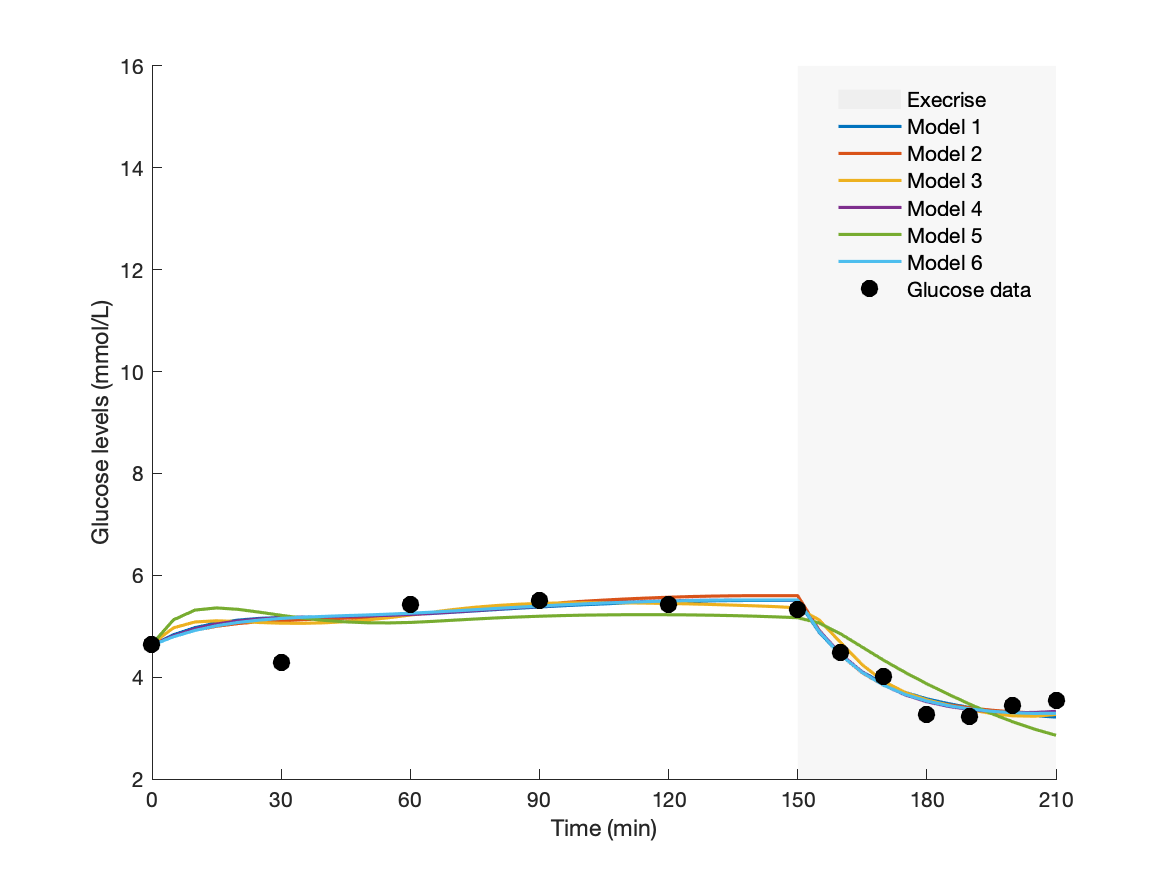** |
| Participant 5**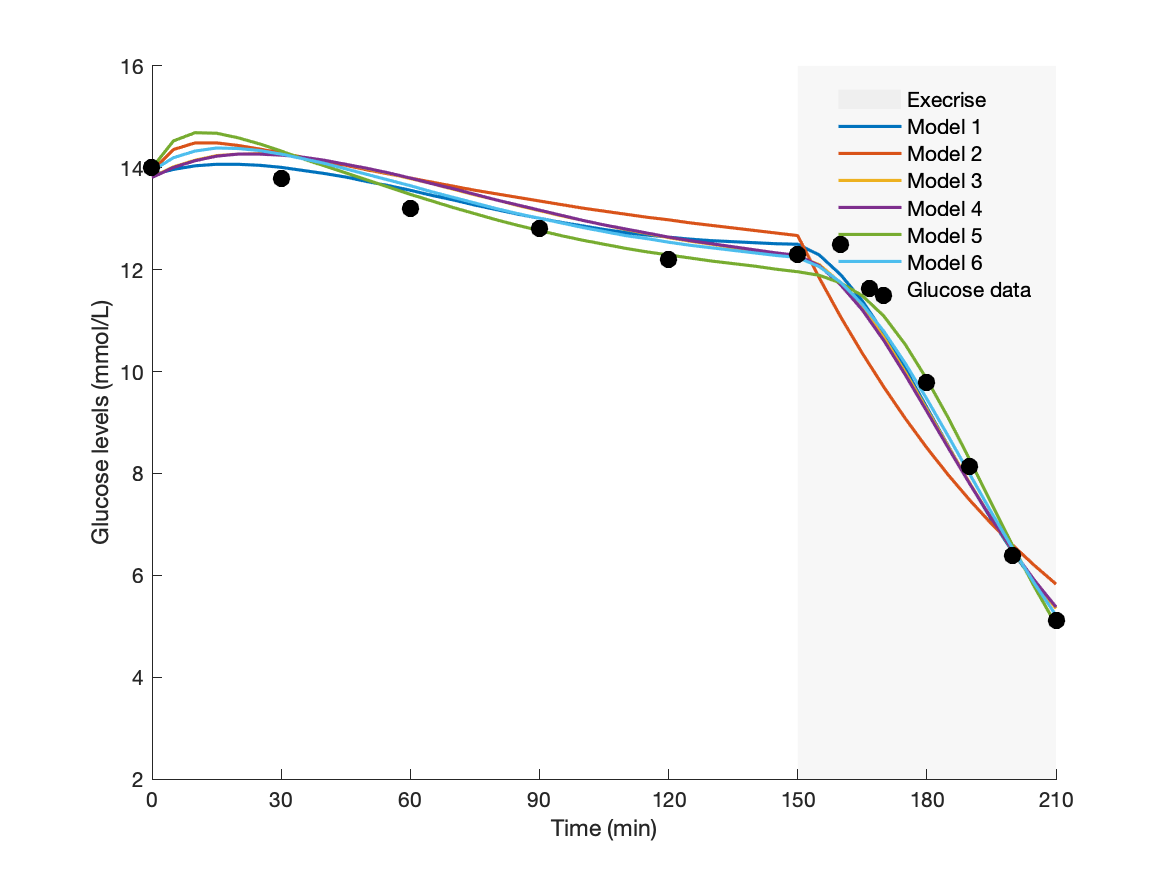** | Participant 6**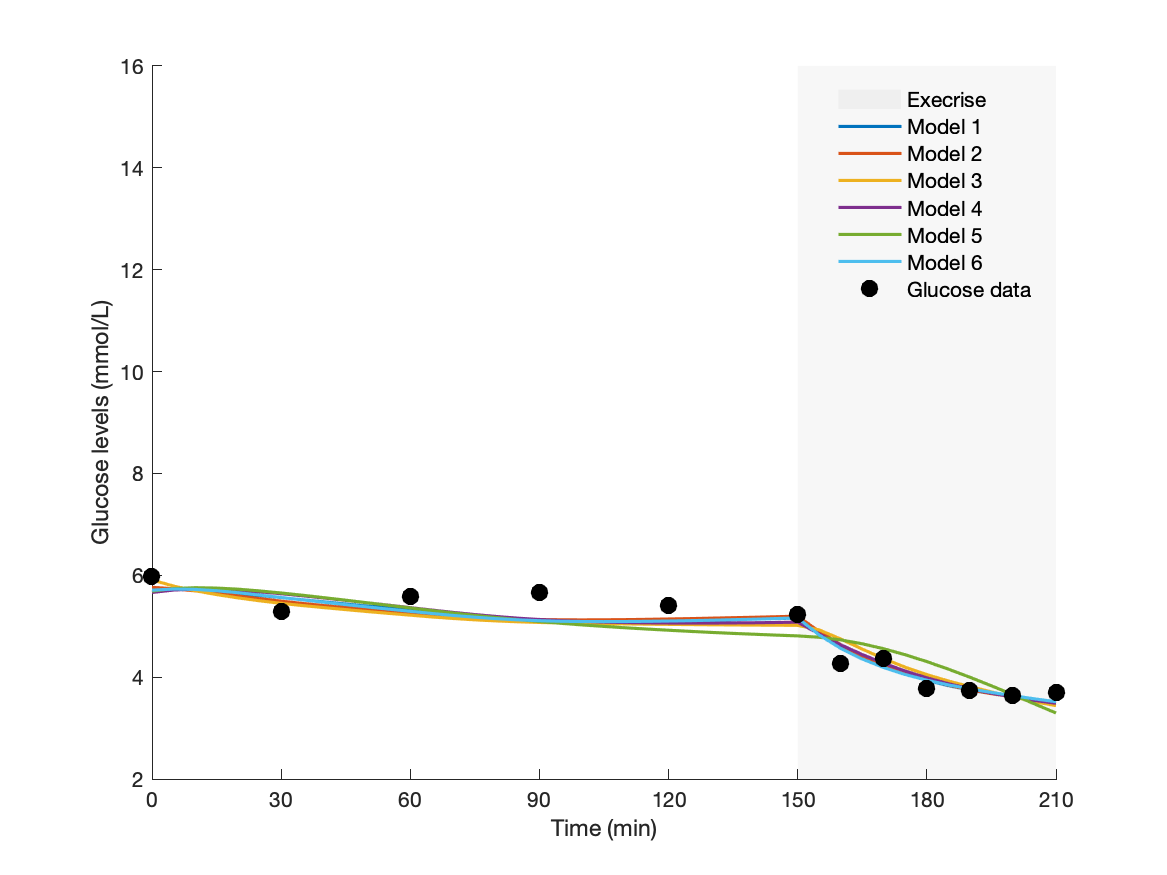** |
| Participant 7**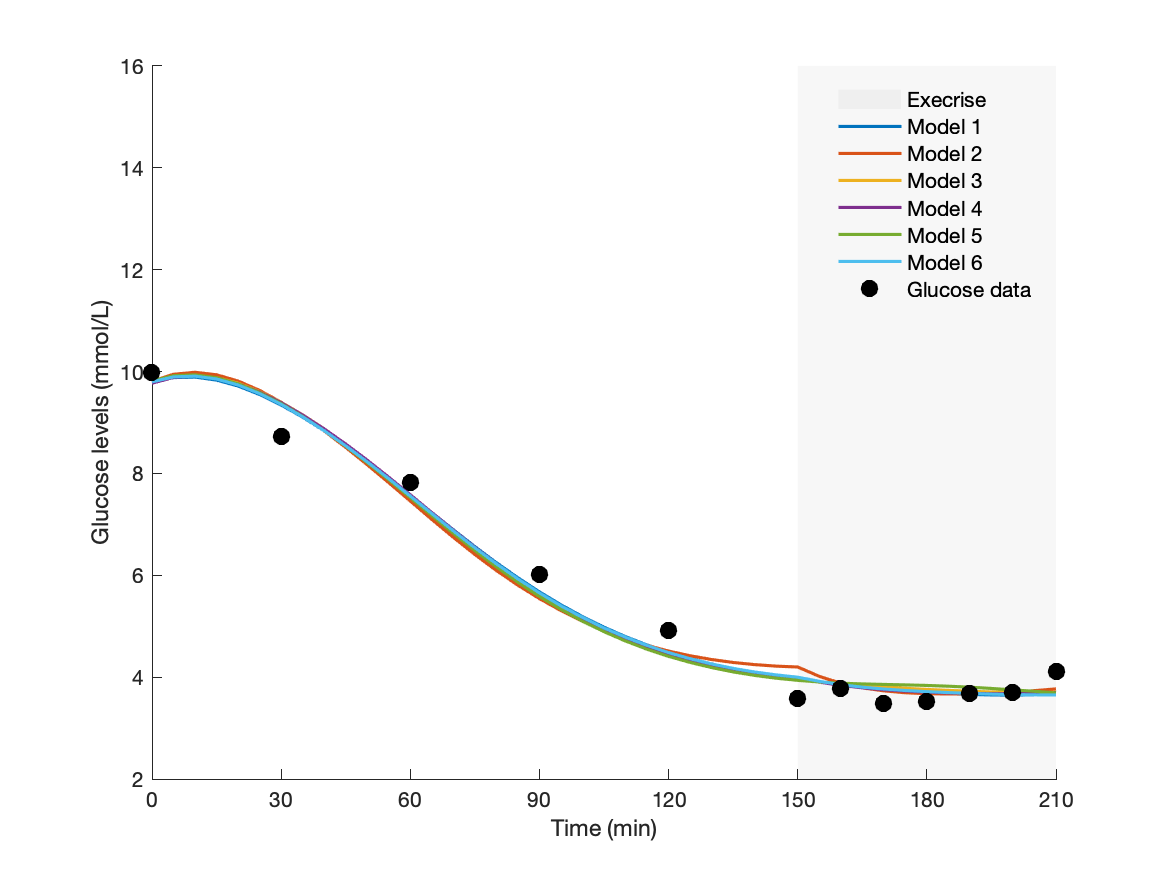** | Participant 8**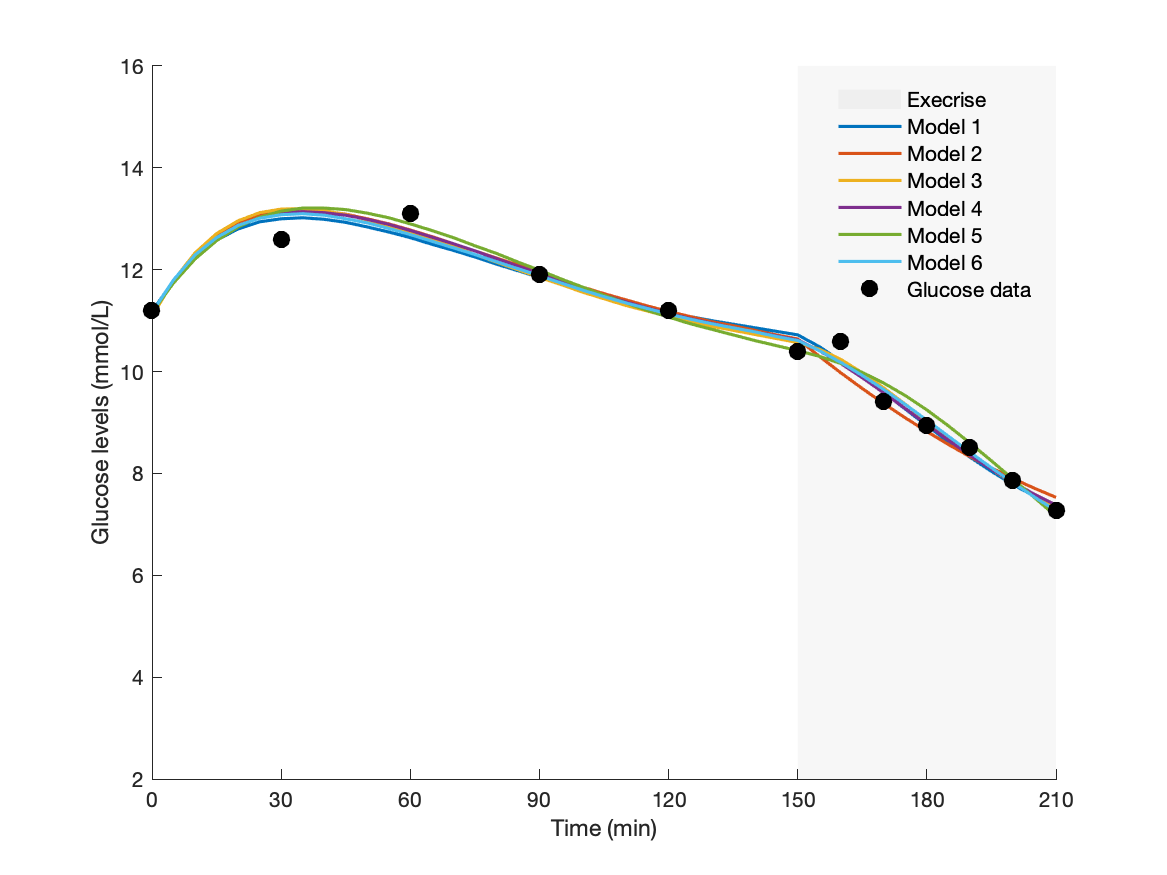** |
| Participant 9**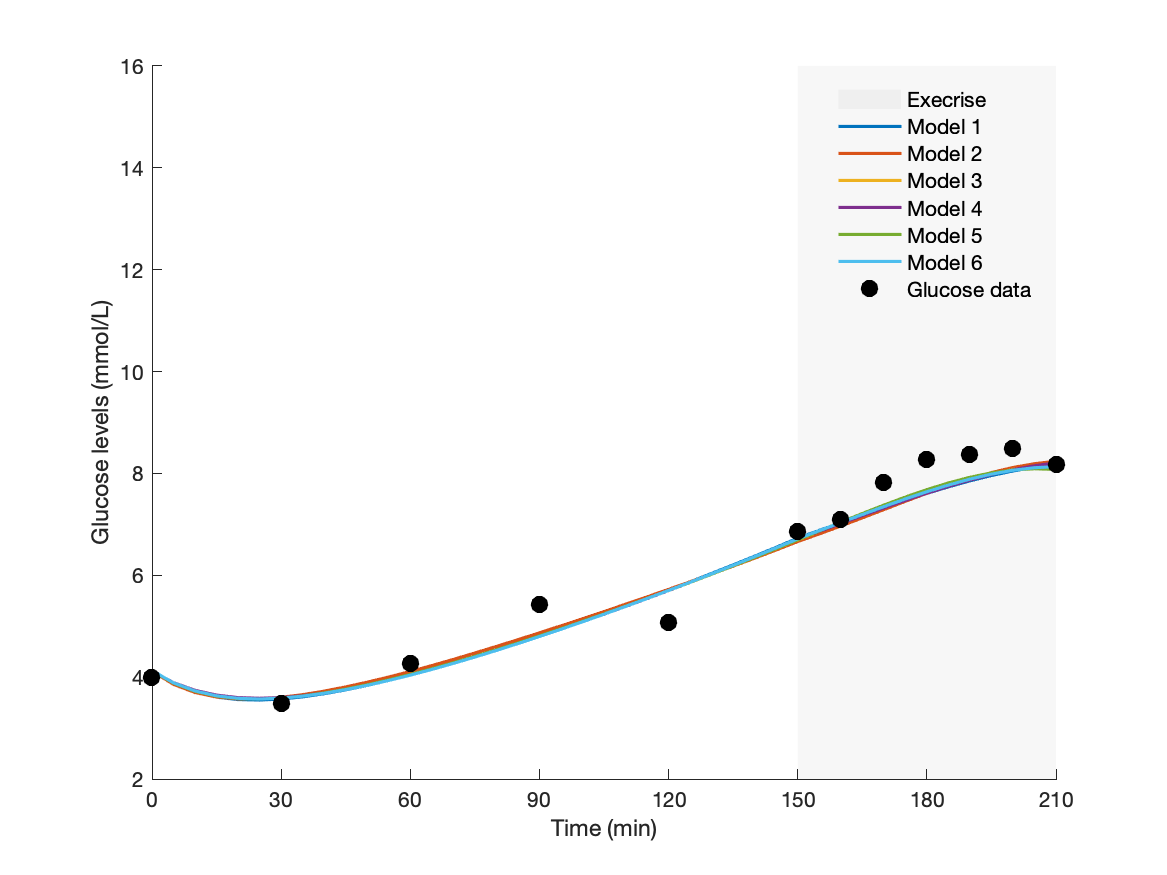** | Participant 10**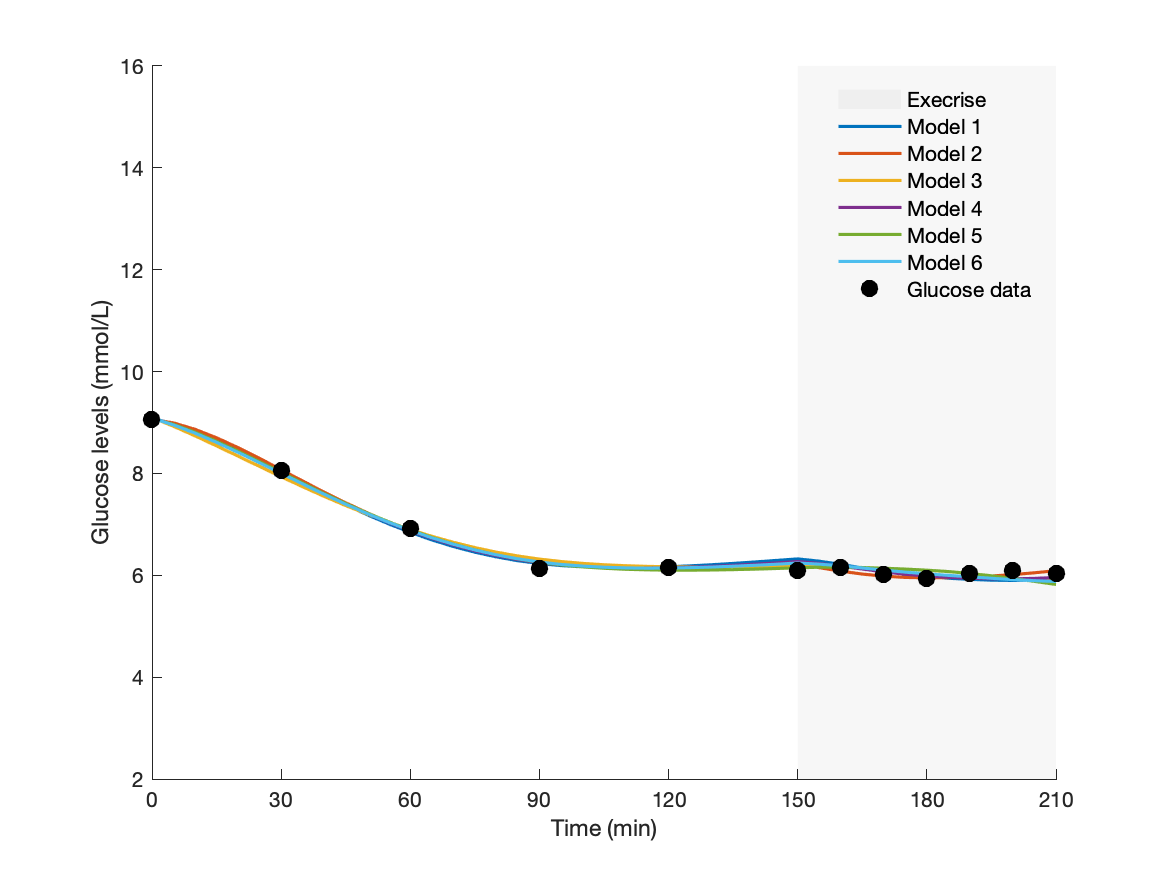** |
| Participant 11  **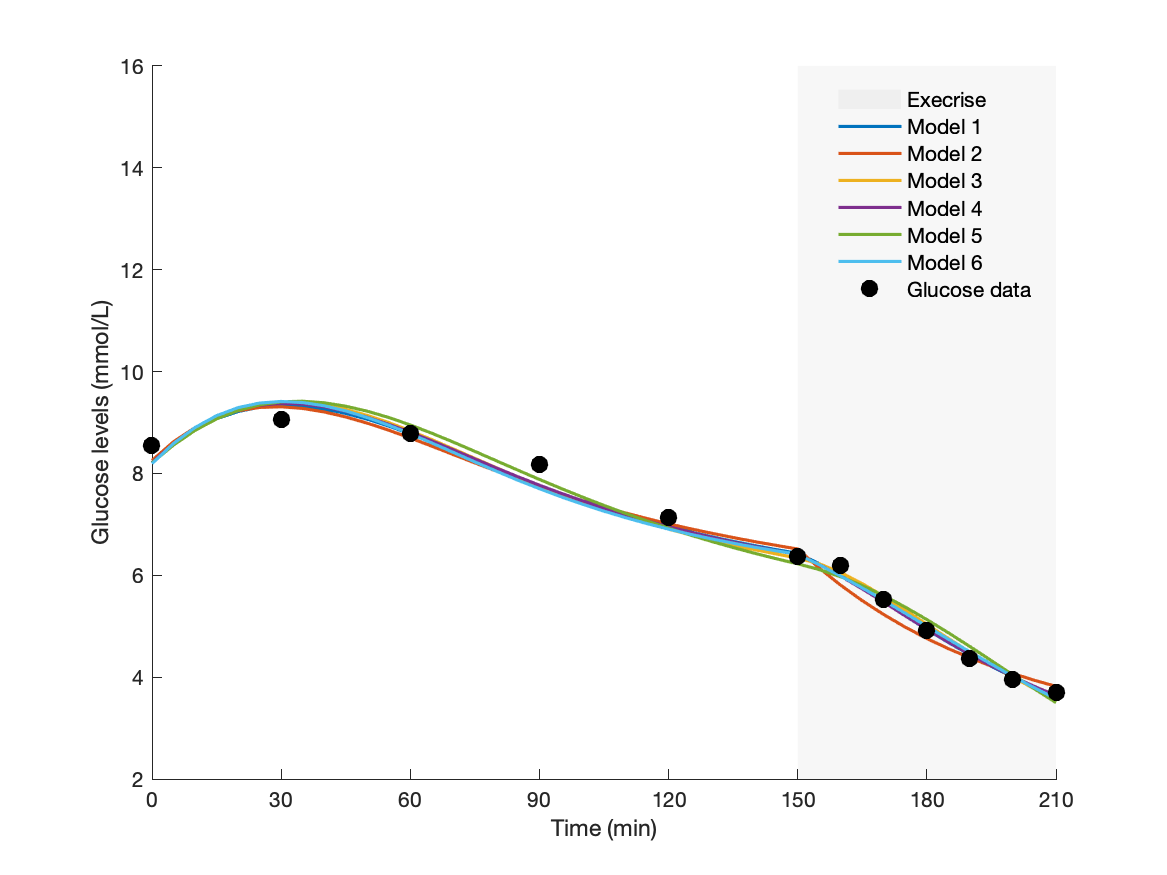** | |

**S1 Fig.** Simulated (models 1-6) vs actual glucose levels (black dots) of participants 1-11. Shaded area is exercise period.

**
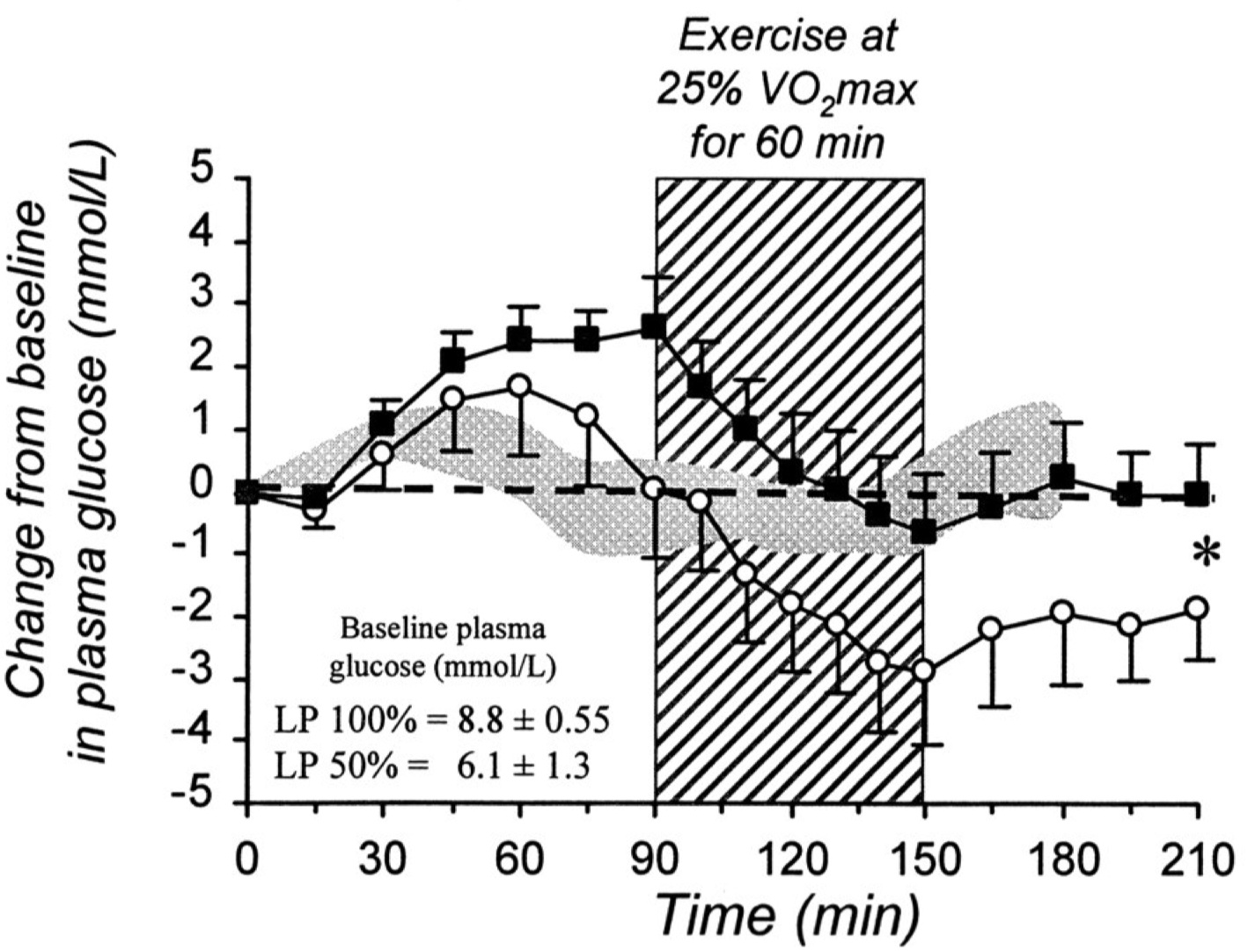

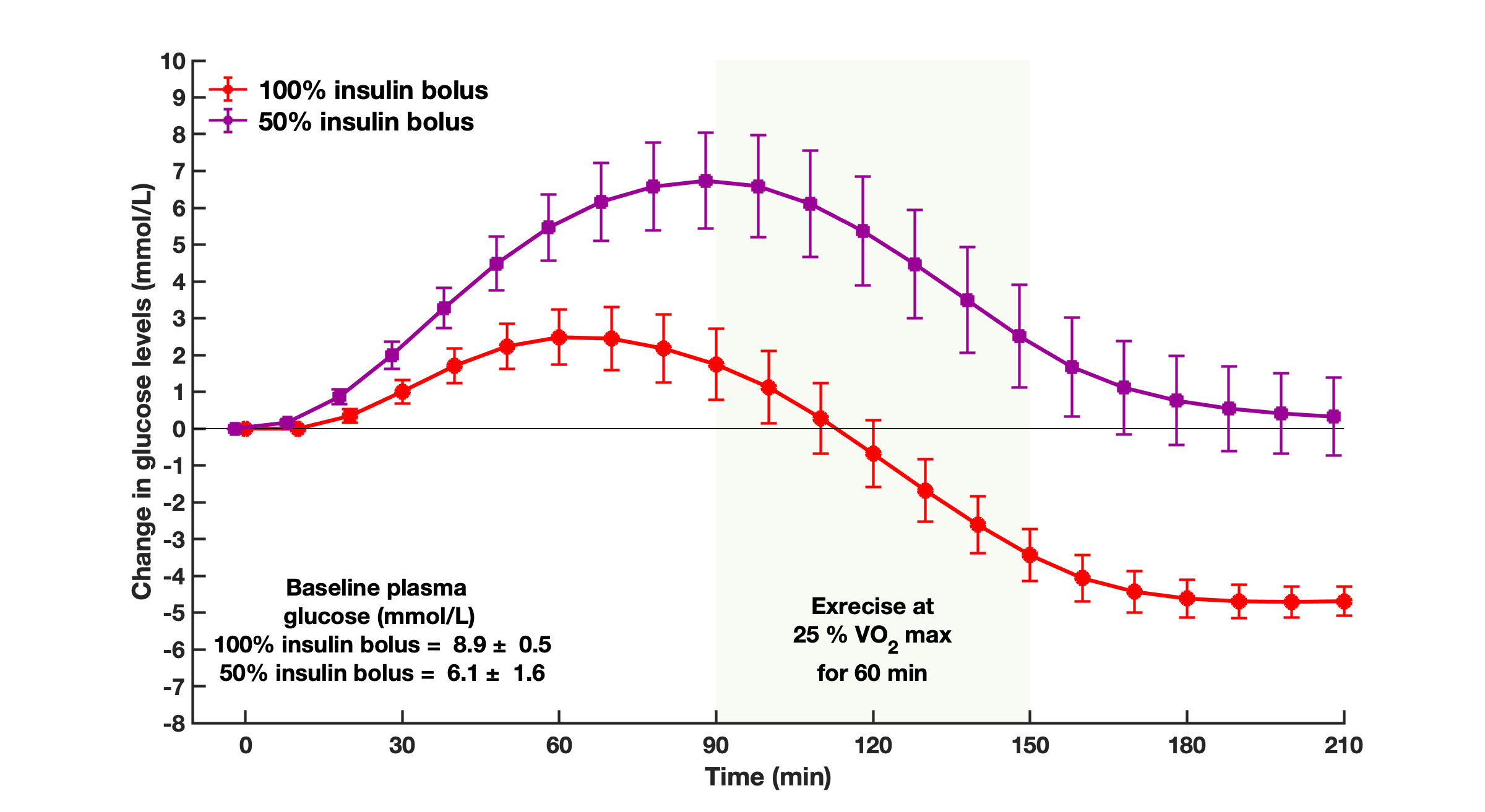
**

**S2 Fig.** Change in glucose levels before, during, and after a 60-minute exercise session at 25% VO2max with 100% and 50% premeal bolus. (Right) results from experimental study (adapted with permission from [8]). (Left) results of simulation experiment.


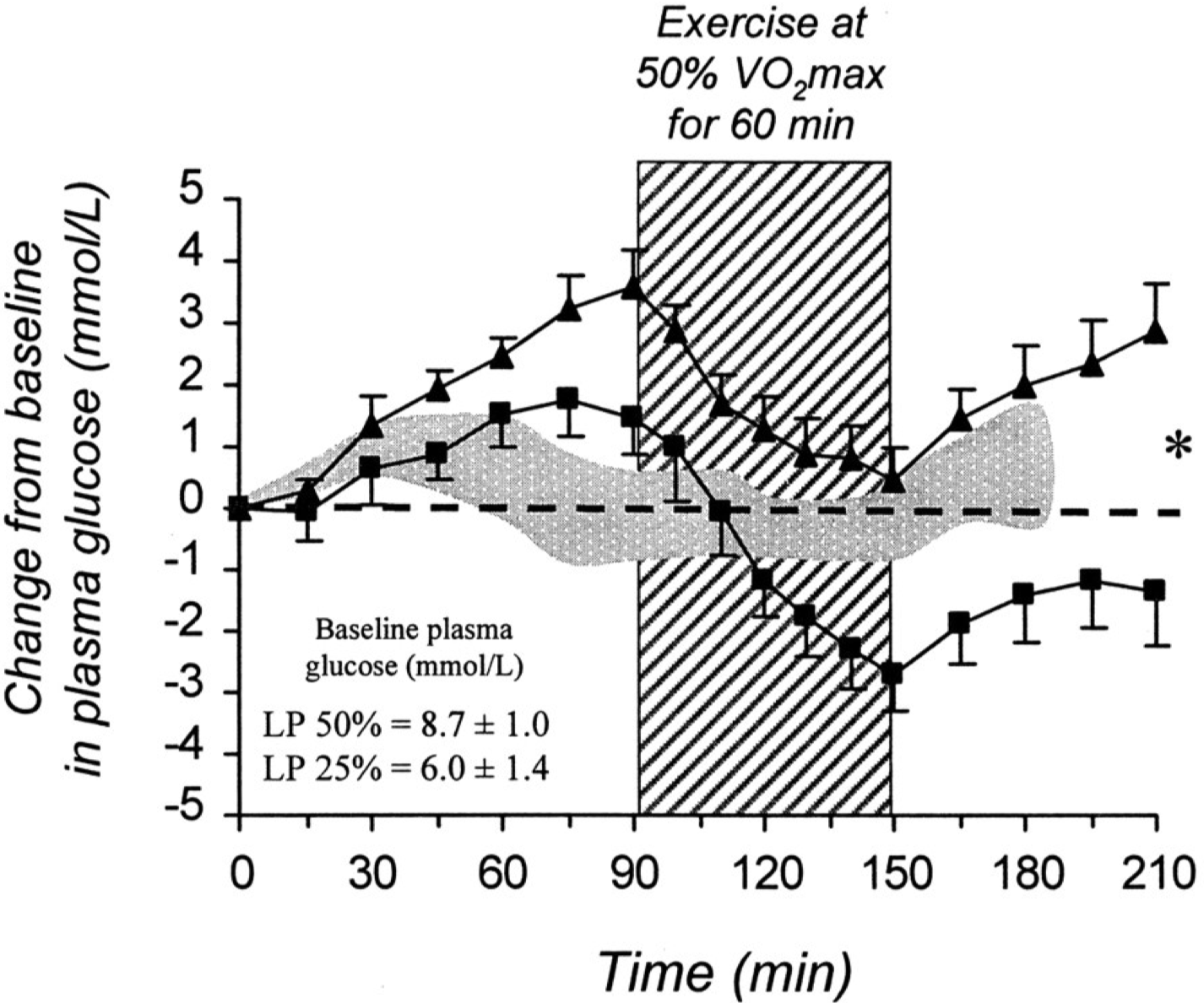

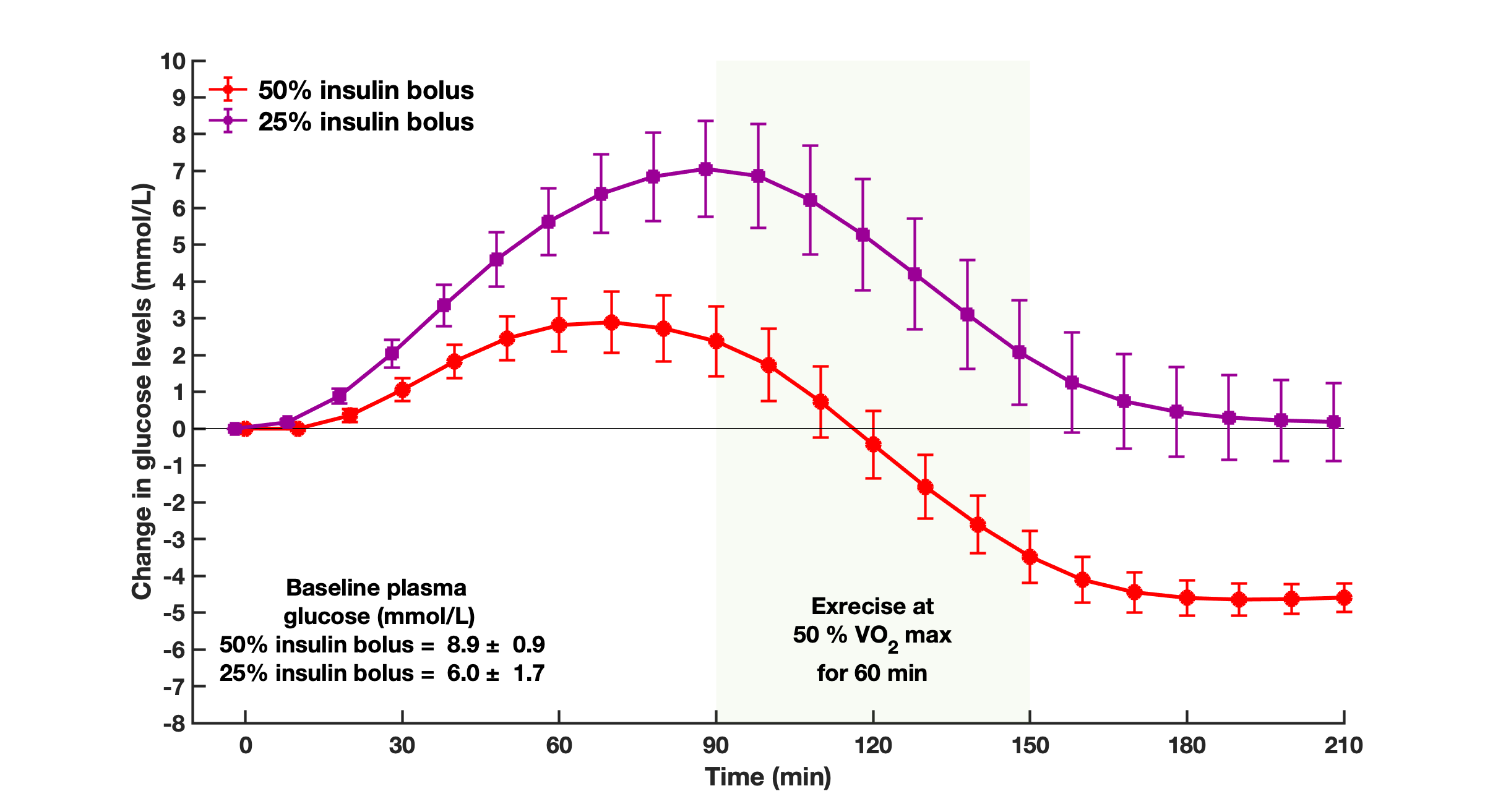


**S3 Fig.** Change in glucose levels before, during, and after a 60-minute exercise session at 50% VO2max with 50% and 25% premeal bolus. (Right) results from experimental study (adapted with permission from [8]). (Left) results of simulation experiment.

**
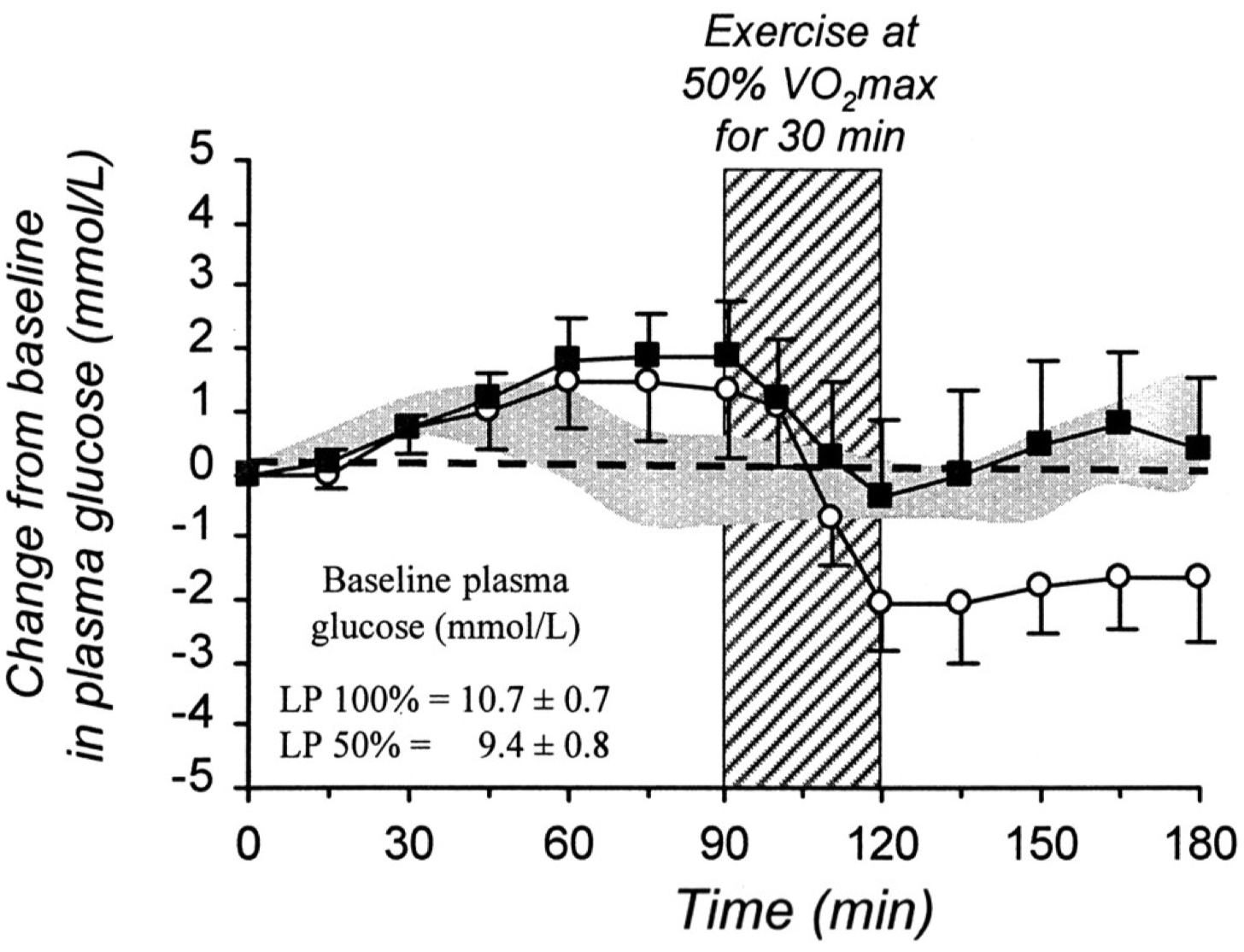

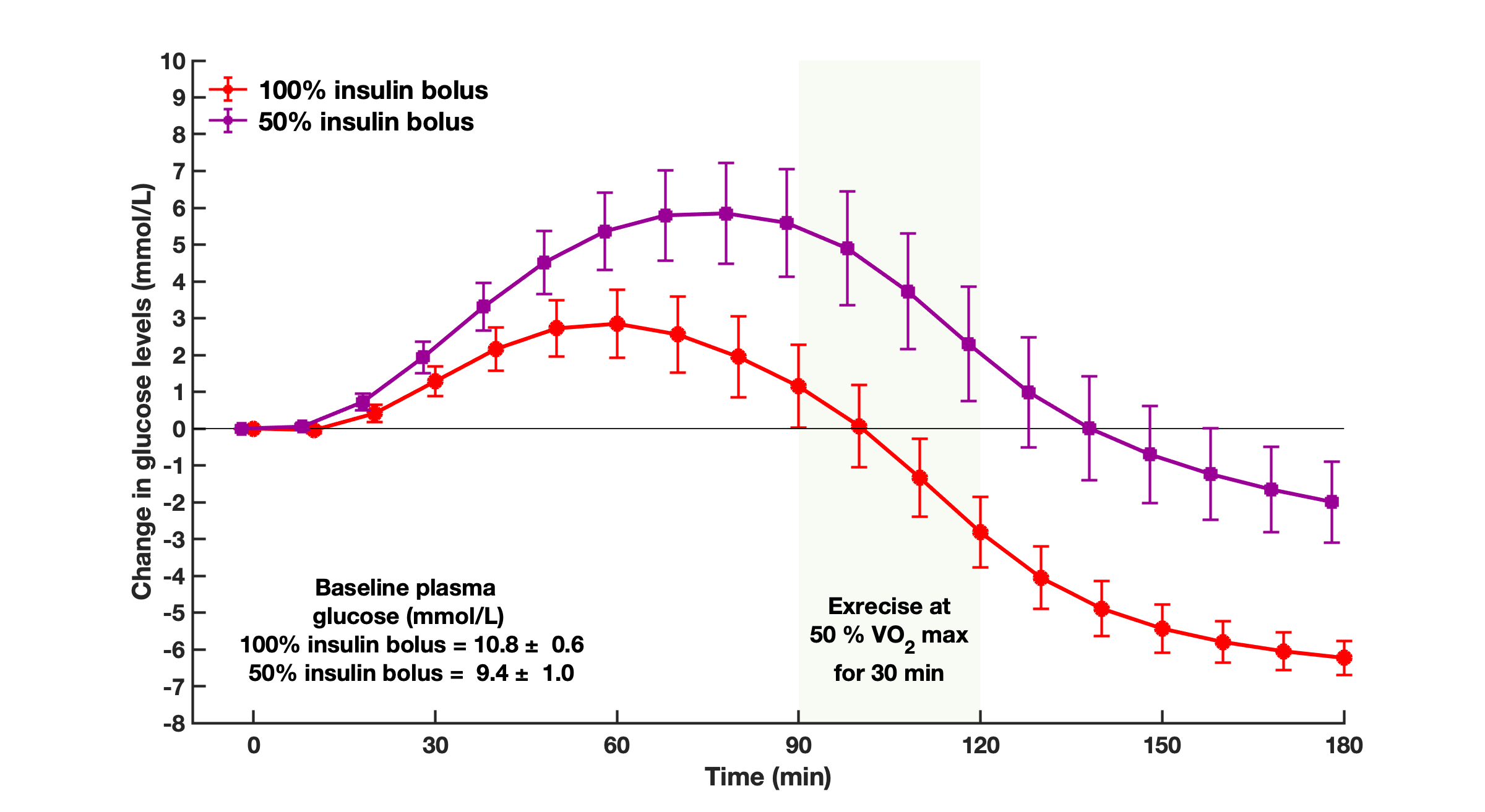
**

**S4 Fig.** Change in glucose levels before, during, and after a 30-minute exercise session at 50% VO2max with 100% and 50% premeal bolus. (Right) results from experimental study (adapted with permission from [8]). (Left) results of simulation experiment.

**
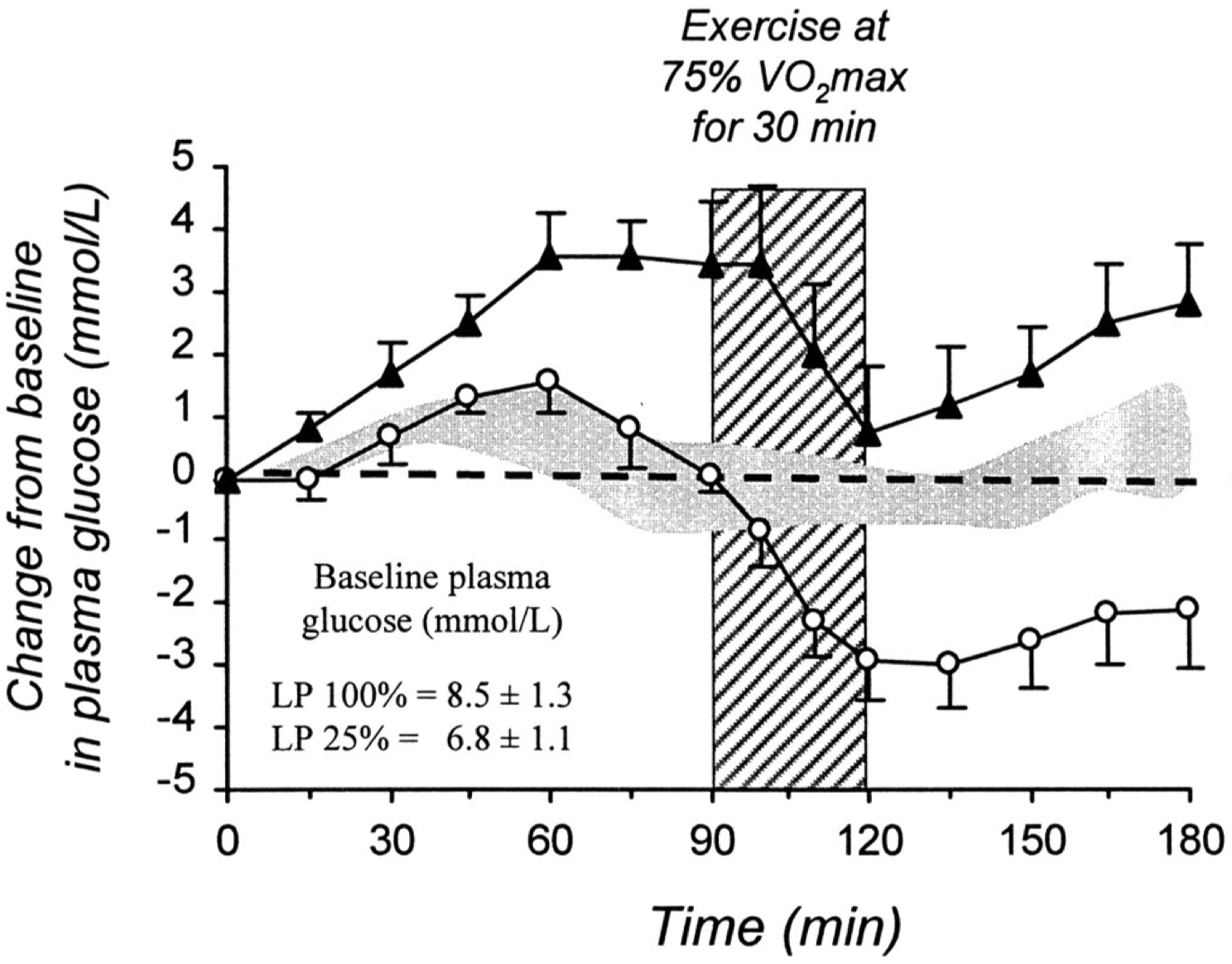

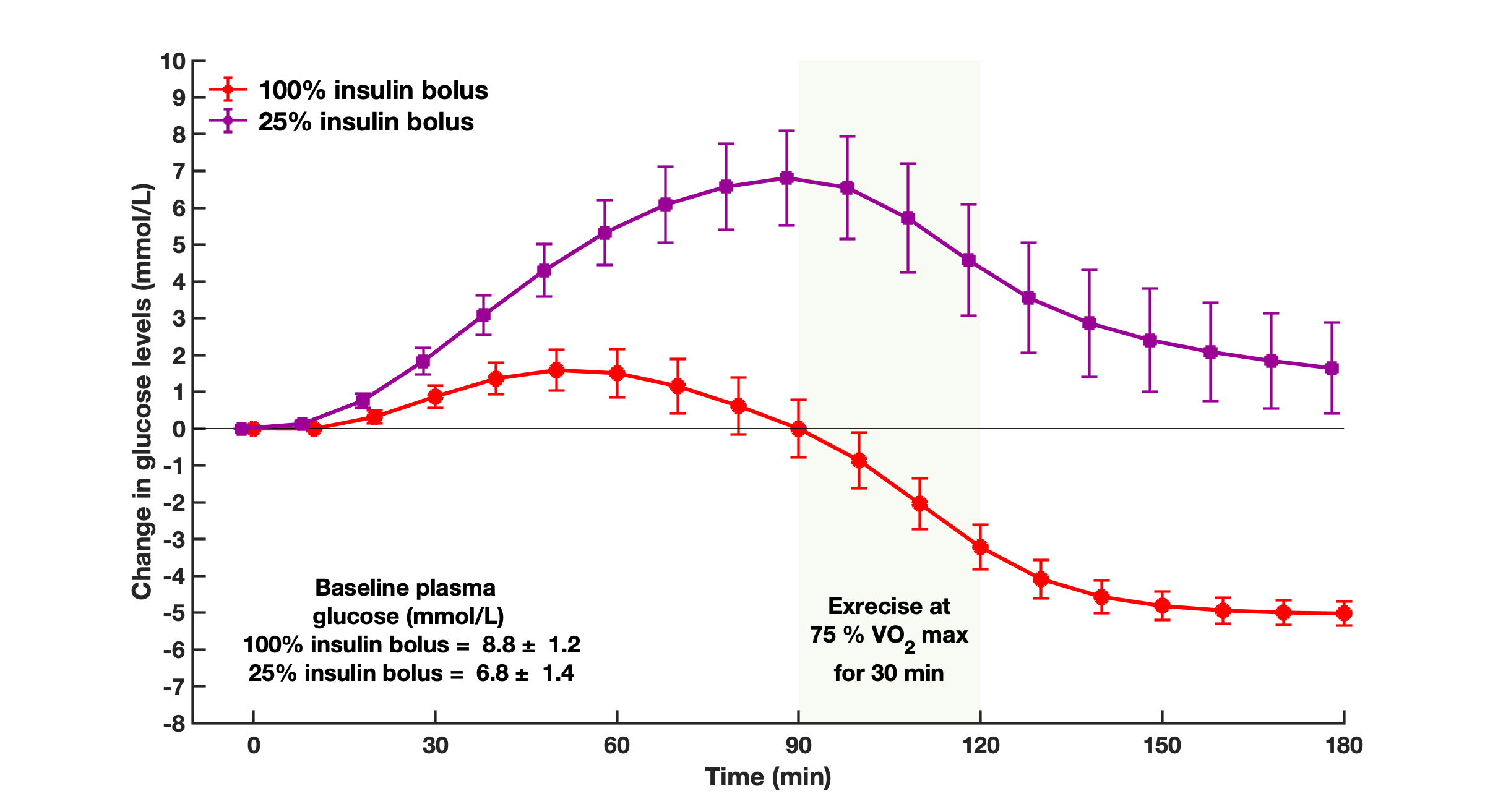
**

**S5 Fig.** Change in glucose levels before, during, and after a 30-minute exercise session at 75% VO2max with 100% and 25% premeal bolus. (Right) results from experimental study (adapted with permission from [8]). (Left) results of simulation experiment.

**
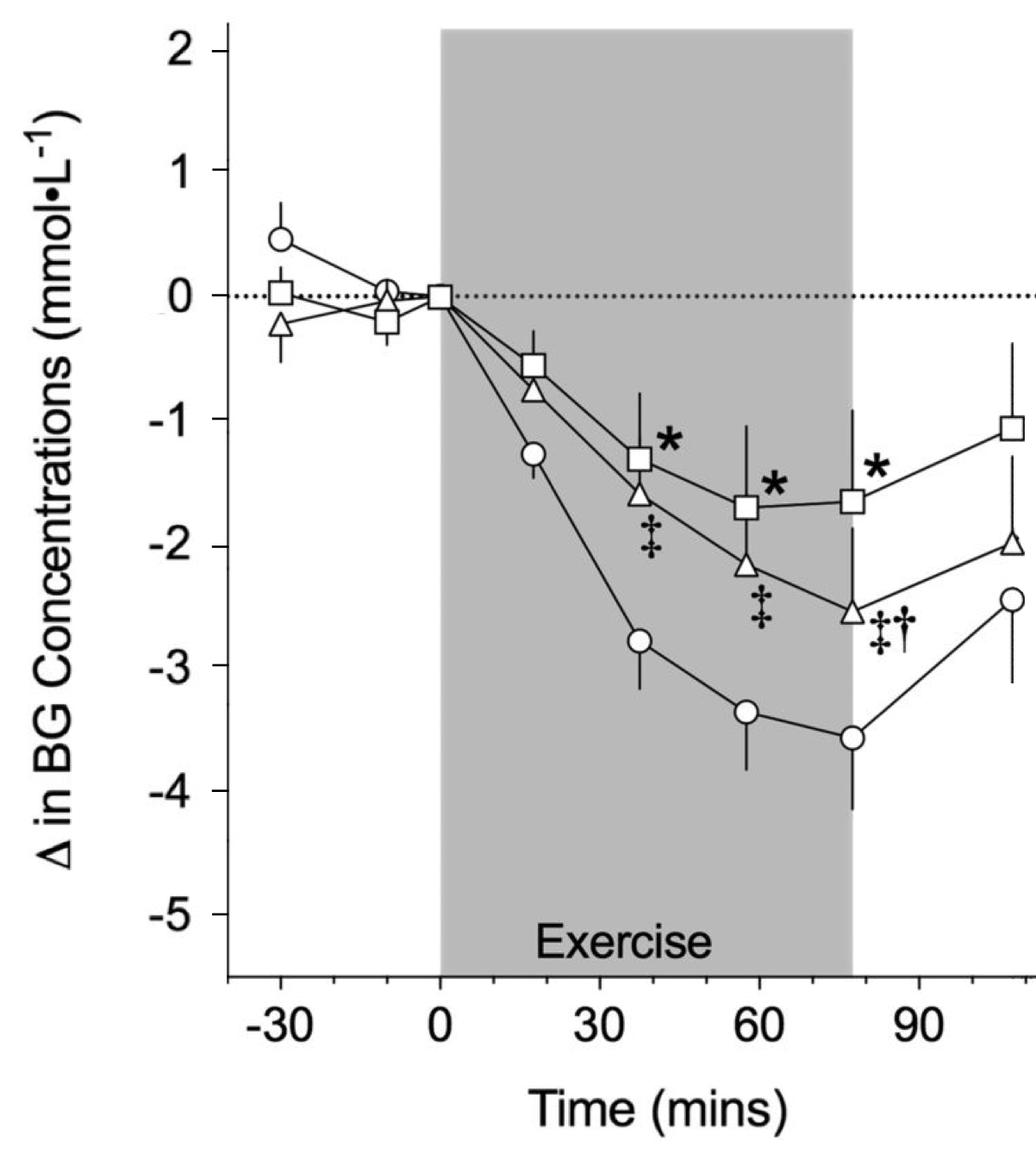

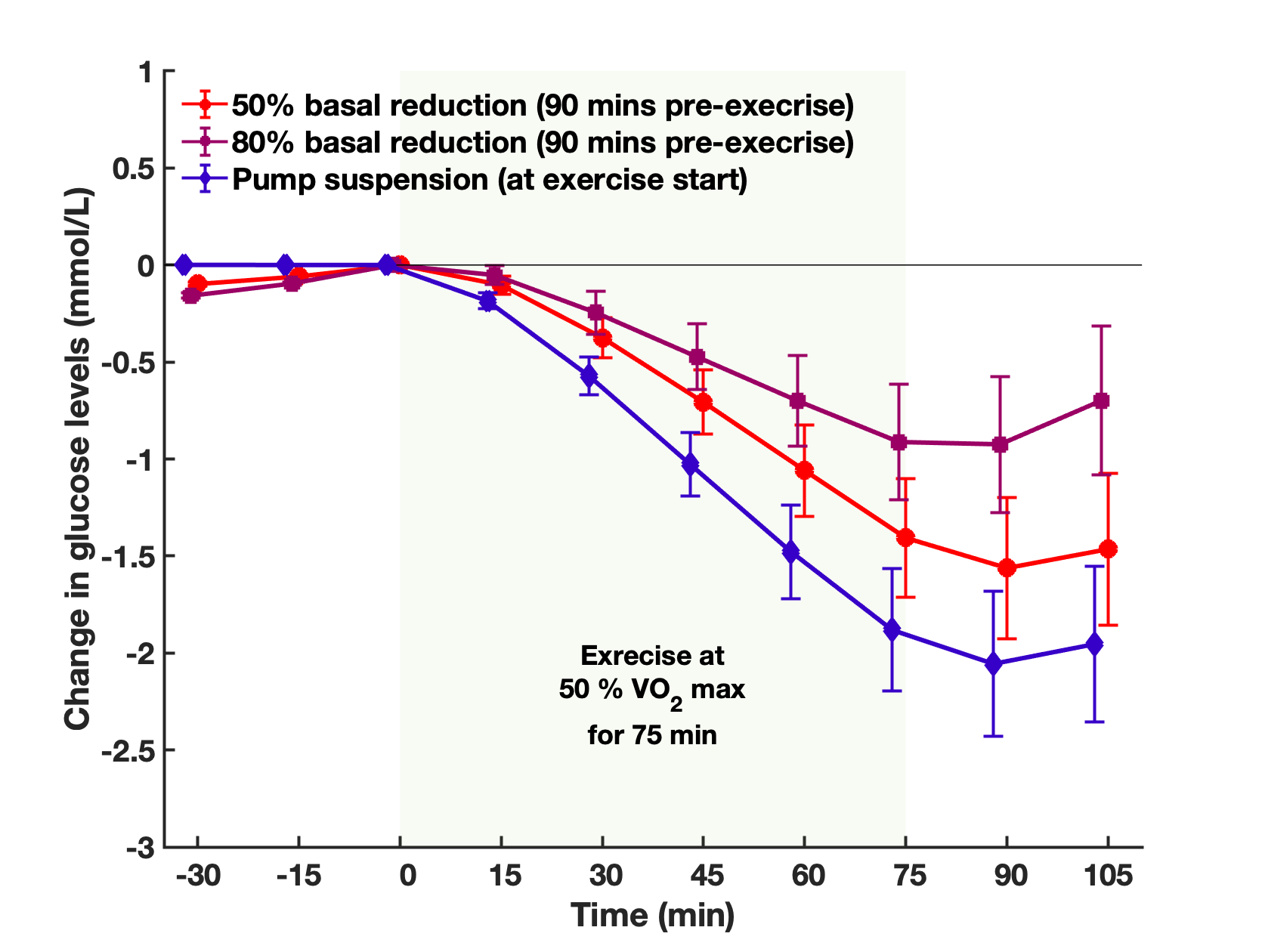
**

**S6 Fig.** Change in glucose levels before, during, and after an exercise session with 50% basal reduction 90-mintues before exercise, 80% basal reduction 90-mintues before exercise, pump suspension at exercise onset. (Right) results from experimental study (adapted with permission from [9]). (Left) results of simulation experiment.

**References**

1. Gilks WR, Richardson S, Spiegelhalter D. Markov chain Monte Carlo in practice: Chapman and Hall/CRC; 1995.

2. Gilks WR, Richardson S, Spiegelhalter DJ. Markov Chain Monte Carlo in Practice. Boca Raton, Fla.: Chapman & Hall; 1998.

3. Lunn DJ, Thomas A, Best N, Spiegelhalter D. WinBUGS - A Bayesian modelling framework: Concepts, structure, and extensibility. Stat Comput. 2000;10(4):325-37. PubMed PMID: ISI:000089242200005.

4. Spiegelhalter D, Thomas A, Best N, Lunn D. WinBugs User Manual. Cambridge, UK: Medical Research Council Biostatistics Unit, 2003.

5. Haidar A, Duval C, Legault L, Rabasa-Lhoret R. Pharmacokinetics of insulin aspart and glucagon in type 1 diabetes during closed-loop operation. Journal of diabetes science and technology. 2013;7(6):1507-12.

6. Worthington DJMI. Minimal model of food absorption in the gut. 1997;22(1):35-45.

7. Hovorka R, Shojaee-Moradie F, Carroll PV, Chassin LJ, Gowrie IJ, Jackson NC, et al. Partitioning glucose distribution/transport, disposal, and endogenous production during IVGTT. American Journal of Physiology - Endocrinology And Metabolism. 2002;282(5):E992-E1007. doi: 10.1152/ajpendo.00304.2001.

8. Rabasa-Lhoret R, Bourque J, Ducros F, Chiasson J-L. Guidelines for premeal insulin dose reduction for postprandial exercise of different intensities and durations in type 1 diabetic subjects treated intensively with a basal-bolus insulin regimen (ultralente-lispro). Diabetes Care. 2001;24(4):625-30.

9. Zaharieva DP, McGaugh S, Pooni R, Vienneau T, Ly T, Riddell MCJDc. Improved open-loop glucose control with basal insulin reduction 90 minutes before aerobic exercise in patients with type 1 diabetes on continuous subcutaneous insulin infusion. Diabetes Care. 2019;42(5):824-31.
